# Interface-guided phenotyping of coding variants in the transcription factor RUNX1

**DOI:** 10.1016/j.celrep.2024.114436

**Published:** 2024-07-04

**Authors:** Kivilcim Ozturk, Rebecca Panwala, Jeanna Sheen, Kyle Ford, Nathan Jayne, Andrew Portell, Dong-Er Zhang, Stephan Hutter, Torsten Haferlach, Trey Ideker, Prashant Mali, Hannah Carter

**Affiliations:** 1 Division of Medical Genetics, Department of Medicine, University of California, San Diego, La Jolla, CA, USA; 2 Bioinformatics and Systems Biology Program, University of California, San Diego, La Jolla, CA, USA; 3 Department of Bioengineering, University of California, San Diego, La Jolla, CA, USA; 4 School of Biological Sciences, University of California, San Diego, La Jolla, CA, USA; 5 Moores Cancer Center, University of California, San Diego, La Jolla, CA, USA; 6 MLL Munich Leukemia Laboratory, Max-Lebsche-Platz 31, 81377 Munich, Germany; 7 Lead contact

## Abstract

Single-gene missense mutations remain challenging to interpret. Here, we deploy scalable functional screening by sequencing (SEUSS), a Perturb-seq method, to generate mutations at protein interfaces of RUNX1 and quantify their effect on activities of downstream cellular programs. We evaluate single-cell RNA profiles of 115 mutations in myelogenous leukemia cells and categorize them into three functionally distinct groups, wild-type (WT)-like, loss-of-function (LoF)-like, and hypomorphic, that we validate in orthogonal assays. LoF-like variants dominate the DNA-binding site and are recurrent in cancer; however, recurrence alone does not predict functional impact. Hypomorphic variants share characteristics with LoF-like but favor protein interactions, promoting gene expression indicative of nerve growth factor (NGF) response and cytokine recruitment of neutrophils. Accessible DNA near differentially expressed genes frequently contains RUNX1-binding motifs. Finally, we reclassify 16 variants of uncertain significance and train a classifier to predict 103 more. Our work demonstrates the potential of targeting protein interactions to better define the landscape of phenotypes reachable by missense mutations.

## In brief

Deciphering distinct effects of single-gene missense mutations is challenging. Ozturk et al. design an interface-guided Perturb-seq library and measure the impact of >100 RUNX1 variants on >40,000 single-cell RNA profiles. The assay identifies functionally distinct groups, demonstrating the potential of scRNA-seq to characterize cellular phenotypes reachable by individual mutations.

## Graphical Abstract

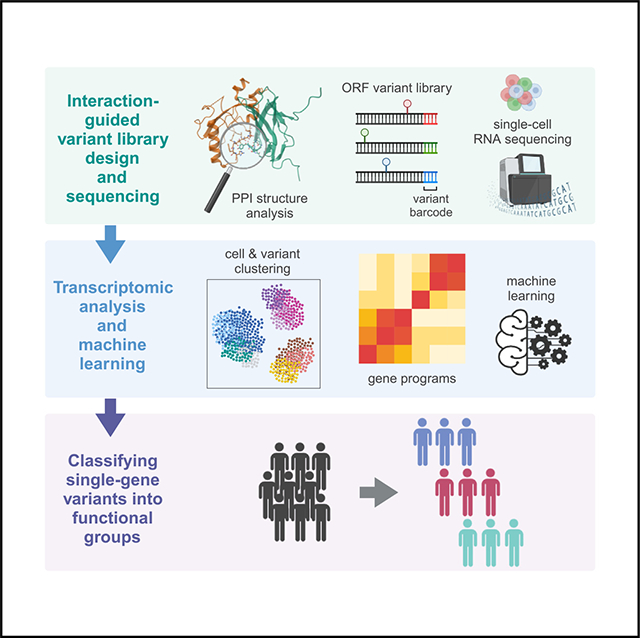

## INTRODUCTION

Cancer is associated with the progressive loss of cell identity and gain of signals promoting inappropriate survival and proliferation. Somatic mutations, particularly in oncogenes and tumor suppressors, alter cellular signaling to promote these changes during tumor development and progression.^1–4^ Complicating matters more, different mutations in the same gene can have different associations with prognosis and therapeutic response. For example, KRAS G13-mutant colorectal tumors, but not G12, are sensitive to cetuximab treatment.^5^ In lung cancer, the G13 mutation is associated with shorter overall survival than the G12 mutation.^6^ In breast cancer, TP53 mutations within DNA-binding motifs have worse prognosis than those outside, but within the motifs, codon 179 mutation and the R248W substitution show significantly poorer prognosis than others.^7^ This highlights the need to develop strategies for studying perturbations at a finer scale than gene knockout or knockdown.

High-throughput mutagenesis is a powerful new tool to probe varying consequences of amino acid substitutions across the length of a protein; however, it is currently limited to specific functional readouts, such as target protein abundance^8^ or functional assays.^9–11^ Studying the effects of genetic perturbations on cellular programs and fitness has been challenging using traditional pooled screens. Approaches such as scalable functional screening by sequencing (SEUSS)^12^ and Perturb-seq^13^ measure the transcriptional consequences of perturbations ranging from whole-gene knockout to amino acid substitutions^14^ in single cells, making it possible to distinguish mutations at the level of cellular programs relevant to cancer progression. SEUSS has been used to study the consequences of functional domain deletions and hotspot mutations to MYC in human pluripotent stem cells.^12^ A Perturb-seq application of driver mutations in KRAS revealed that their impact spans a continuum of function not predicted solely by frequency in cancer cohorts.^14^ While providing greater functional insight, these sequencing-based methods are not yet scalable to exhaustive mutagenesis, necessitating the selection of target mutations.

Individual proteins often have multiple functions, mediated through interaction with different binding partners. Somatic mutations in driver genes are overrepresented at interaction interfaces,^15–19^ suggesting that examining the consequences of perturbing distinct protein interfaces could provide a useful abstraction of phenotypic space reachable by individual amino acid substitutions. To explore this hypothesis, we focused on the Runt-related transcription factor 1 (RUNX1), part of the core-binding factor (CBF) heterodimeric complex, consisting of the DNA-binding RUNX1 and non-DNA-binding CBFB proteins. RUNX1 is characterized by a highly conserved 128-amino acid residue Runt domain, responsible for both binding to DNA^20–23^ and heterodimerization with CBFB,^23–25^ which increases its DNA-binding affinity^26–30^ and occurs at distinct, non-overlapping sites within the domain.^24^ A variety of transcriptional co-regulatory proteins also bind RUNX1 to activate or repress transcription,^31–38^ some through the Runt domain,^39–43^ suggesting that they may compete with one another for RUNX1 interaction.^42^

RUNX1 is required for definitive hematopoiesis.^20,44–46^ It plays an important role in T lymphocyte development and lineage specification^47^ and megakaryocyte differentiation,^47,48^ and it is implicated in erythroid cell differentiation.^46,49,50^ Mutations in RUNX1 are frequently observed in hematopoietic disorders such as acute myeloid leukemia (AML), lymphoid leukemias, myelodysplasias (MDSs), and blast crisis chronic myelogenous leukemia (CML),^51–54^ and less commonly observed in breast cancer.^55,56^ In addition, RUNX1 haploinsufficiency is responsible for familial platelet disorder with predisposition to AML.^57–59^

As RUNX1 is a pioneer transcription factor and master regulator implicated in multiple cancer types, we hypothesized that mutations affecting its interactions with transcriptional co-factors would manifest as changes to expression of RUNX1 target genes, resulting in functional diversity in cancer. We designed a library of 117 variants, with the potential to perturb distinct RUNX1 interactions and implicate different aspects of the RUNX1 regulon, including wild-type (WT) and loss-of-function (LoF) controls as a frame of reference for functional impact. We applied SEUSS to overexpress the library in myelogenous leukemia cells and analyzed single-cell transcriptional readouts to identify functionally distinct groups of RUNX1 mutations, characterize their effects on cellular programs, and study implications for cancer.

## RESULTS

### An interface-guided Perturb-seq assay for coding variant phenotyping of RUNX1

**Figure 1.**
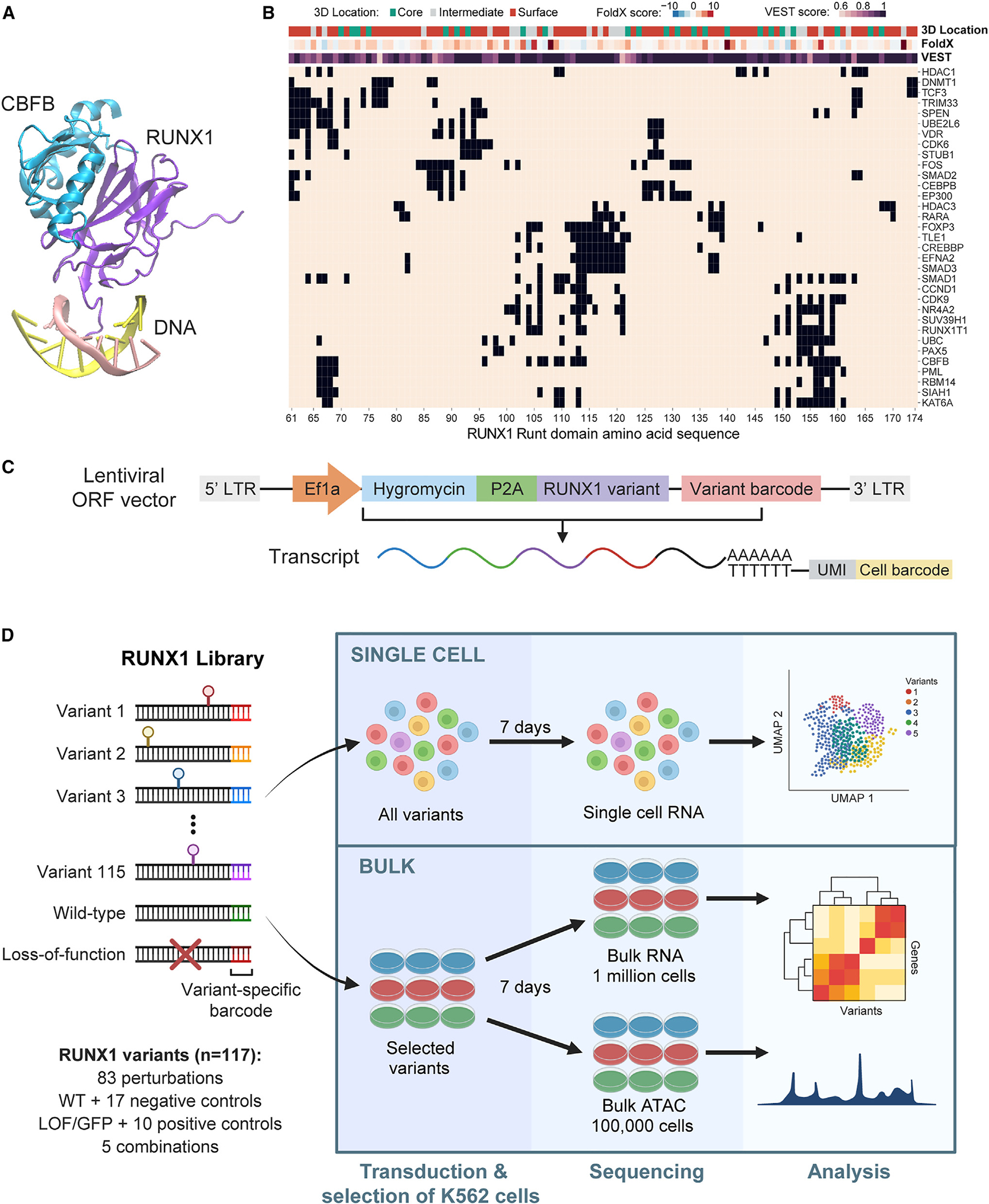
Interface-guided Perturb-seq assay for coding variant phenotyping of RUNX1 (A) The 3D crystal structure of transcription factor CBF, consisting of RUNX1 Runt domain (purple) and CBFB (blue), interacting with DNA (yellow and pink strands) (PDB: 1h9d). (B) Amino acid residue map of RUNX1 Runt domain (columns). In each row, RUNX1 interface residues involved in interaction with each protein partner (rows) are highlighted by black. Rows are hierarchically clustered. Top: residue 3D location annotations (core, intermediate, surface), VEST and FoldX scores of the most damaging mutations targeting each residue. Color darkness indicates mutation impact: damaging (VEST) or destabilizing (FoldX). (C) Lentiviral ORF vector containing RUNX1 variant (WT, mutated, or GFP) and 12-bp variant-specific barcode sequence. (D) Experimental and computational overview: ORF variant library design, transduction, scRNA-seq of all 117 library elements, bulk RNA-seq, and ATAC-seq of 12 selected elements; computational analysis.

While somatic mutations in RUNX1 span the entire gene, the most recurrent mutations cluster in the Runt domain. We used protein structures (Figure 1A) and template-based docking^60^ to identify 83 amino acid residues in the Runt domain involved in physical interactions with at least one of 33 protein partners with structural data (Figures 1B and S1) (STAR Methods). These were used to design an open reading frame (ORF) mutation library to assess the impact of perturbation of various RUNX1 interactions. We used the RUNX1B isoform, the canonical sequence in UniProt,^61^ but we provided mappings to the RUNX1C isoform, more commonly used by mutation databases (Table S1).

For each of the 83 residues, we identified amino acid substitutions that would maximally perturb function based on variant effect scoring tool (VEST) pathogenicity scores^62^ and FoldX folding free energy predictions,^63^ while prioritizing substitutions observed in tumors from the Catalogue Of Somatic Mutations In Cancer (COSMIC).^64^ To provide a frame of reference for functional impact, we included WT RUNX1 and LoF control constructs (RUNX1 replaced with the GFP), 17 negative controls (expected to be indistinguishable from WT) consisting of 10 silent and 7 predicted neutral (based on VEST scores) mutations, and 10 positive controls (expected to mimic LoF) comprising 5 truncating and 5 core mutations. To evaluate the mutation combinatorial impact, we generated 5 combinations, bringing the total to 117 library elements (Table S1).

We used SEUSS^12^ to overexpress the mutant RUNX1 library in K562, a CML cell line with WT RUNX1.^65^ We generated a clonal K562 cell line with doxycycline-inducible CRISPRi knockdown of endogenous RUNX1 (iRUNX1-KD K562) and measured 67% reduction in RNA and 72% reduction in protein expression by qRT-PCR and western blot, respectively (Figures S2A–S2C). Our RUNX1 variant ORF overexpression library was generated from a lentiviral vector modified to contain a hygromycin resistance gene downstream of the EF1a promoter, followed by a P2A peptide motif, the RUNX1 variant (WT, mutated, or GFP as LoF control), and a 12-bp barcode sequence unique to each variant for identification after single-cell transcriptome sequencing (Figure 1C). The iRUNX1-KD K562 cells were transduced with the pooled variant library at a low (~0.3) MOI to ensure that each cell received a single construct and then were grown with hygromycin to select those carrying constructs. Cells were split into two populations, one treated with doxycycline (dox) to induce repression of endogenous RUNX1 (dox), the other not (nodox). At day 7 post-transduction, single-cell RNA libraries were prepared and sequenced, with the remaining cells being maintained until day 14 for fitness screening (Figure 1D). We confirmed RUNX1 protein overexpression for the WT control construct relative to the GFP/LoF construct as well as the dox-inducible repression of endogenous RUNX1 protein in both contexts via western blot (Figures S2D and S2E) (STAR Methods).

We generated single-cell transcriptional profiles for 86,120 cells using 10X Genomics Chromium,^66^ 48.4% of which contained detectable variant barcodes assigned to a single variant only. After quality control (QC) filtering, we recovered 40,522 high-quality single-variant cells covering 112 of 117 assayed variants for downstream analysis (STAR Methods). A total of 20,878 cells were from the pool treated with dox, and although significantly correlated (*r* = 0.96, *p* = 1.3e–18), they showed larger effect sizes associated with RUNX1 mutations relative to cells still expressing endogenous RUNX1 (nodox) (Figure S3). Therefore, we focused our remaining analysis on the 20,878 high-quality cells without endogenous RUNX1 (median 136 cells per variant; Figure S4A; Table S2). LoF (361 cells) and positive control variants (median 244 cells per variant) generated significantly higher numbers of cells in comparison to WT (127 cells) and negative controls (median 63 cells per variant) (*r* = 0.85, *p* = 7.5e–9) (Figure S4B), consistent with reports that reduction or loss of RUNX1 activates cell proliferation,^67,68^ although this may differ in other contexts.^69,70^ Mutation combinations generated even more cells than the LoF control (Figure S4B).

### Unsupervised transcriptome-based clustering of cells and variants

**Figure 2.**
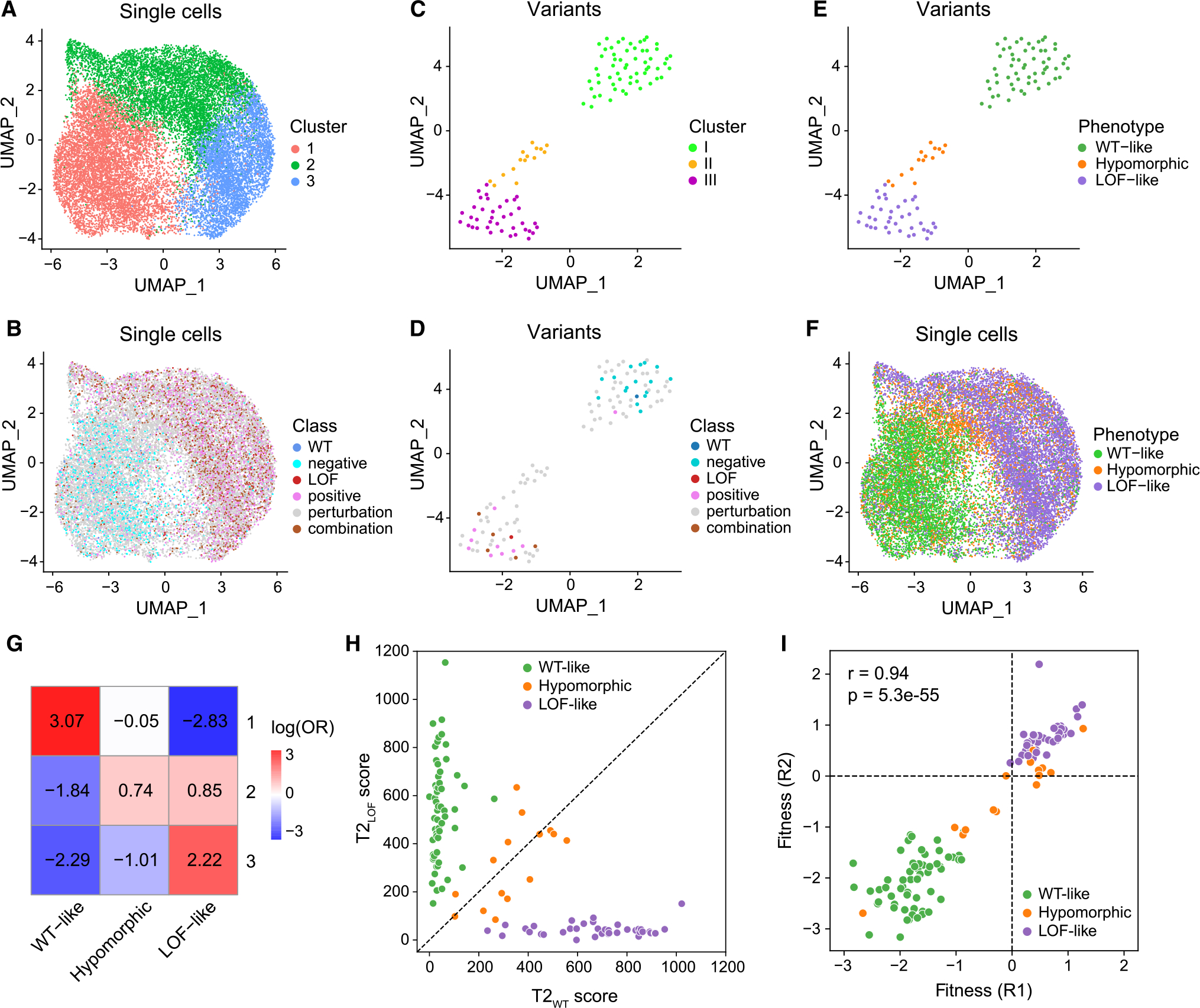
Unsupervised analysis of RUNX1 variant transcriptional effects informs WT-like, LoF-like, and hypomorphic variants (A and B) UMAP embedding of single cells, colored by (A) unsupervised clusters and (B) variant classes. Cell-cycle effects are regressed out. (C and D) UMAP embedding of variants, constructed from mean expression across cells, colored by (C) unsupervised clusters and (D) variant classes. (E and F) UMAP embedding of (E) variants or (F) single cells carrying those variants, colored by variant functional designations (phenotype: WT-like, LoF-like, or hypomorphic) for unsupervised clusters in (C). (G) Enrichment of single cells with assigned phenotypes from (F) for unsupervised clusters in (A). Positive and negative values indicate enrichment and depletion, respectively. (H) Variant T2 scores when compared to WT (x axis) or LoF (y axis) controls. (I) Variant fitness scores from 2 biological replicates (R1: replicate 1, R2: replicate 2; Pearson’s r = 0.94, *p* = 5.3e-55).

We reasoned that variant function could be assessed via transcriptome-based clustering of cells, such that cells harboring variants with similar effects on RUNX1 targeting group together, while those with distinct effects separate. Here, cells carrying the WT or LoF control constructs provide a frame of reference for designating functional impact, and cells clustering away from the WT are considered to be harboring “functional” variants. After regressing out cell-cycle effects (Figures S5A and S5C), we performed unsupervised clustering of single-cell gene expression profiles, which supported three clusters (Figure 2A). Cluster 1 harbored the majority of cells expected to be functionally WT (WT construct: log(odds ratio [OR]) = 1.69, *p* = 7.3e–19, and negative controls: log(OR) = 2.69, *p* = 6.7e–301), whereas LoF construct and positive control mutations were most enriched in clusters 2 and 3 (LoF: log(OR) = 0.28, *p* = 0.01, positives: log(OR) = 0.34, *p* = 9.6e–15, for cluster 2; LoF: log(OR) = 0.87, *p* = 2.2e–15, positives: log(OR) = 0.88, *p* = 2.5e–86 for cluster 3) (Figures 2B and S5D). Although cells containing perturbation variants were more enriched in cluster 1 overall (log(OR) = 0.62, *p* = 3.3e–82; Figure S5D), certain variants concentrated in cluster 2 (e.g., S114L, log(OR) = 1.28, *p* = 4.7e–7) or 3 (e.g., T169I, log(OR) = 1.39 *p* = 1.3e–15) (Figure S5E; Table S3).

Next, we performed unsupervised clustering of variants using their mean gene expression profiles across cells, which again suggested three groups (Figure 2C). Group I included the WT construct and all negative control variants. Group III contained the LoF construct, and the majority of positive controls (8 of 10) expected to generate a non-functional protein (Figure 2D). While most perturbation variants also fell into these groups (I: 41 variants, III: 24 variants), reflecting expression profiles similar to WT or LoF, the separate assignment of 14 variants to group II suggested a partial loss of RUNX1 function, distinct from LoF or WT activity. Accordingly, we labeled variants in these groups as “WT-like,” “LoF-like,” and “hypomorphic” (Figure 2E). Revisiting the single-cell space (Figure 2F), cells carrying WT-like and hypomorphic variants largely separated into clusters 1 (log(OR) = 3.07, *p* < 2.2e–308) and 2 (log(OR) = 0.74, *p* = 9.9e–89), whereas cells harboring LoF-like variants concentrated in both clusters 2 (log(OR) = 0.85, *p* = 5.7e–172) and 3 (log(OR) = 2.22, *p* < 2.2e–308) (Figure 2G).

To further investigate hypomorphic variants, we quantified differences in the distributions of single-cell transcriptional profiles of each variant against WT or LoF controls with Hotelling’s T-squared test statistic (T2 score),^71^ where higher scores indicate a higher deviation from the control (STAR Methods). This statistical analysis revealed that all WT-like variants are indistinguishable from the WT control via small T2 scores relative to WT (T2_WT_), but high scores relative to LoF (T2_LoF_) (*p* < 0.05). Similarly, all LoF-like variants are indistinguishable from the LoF control via small T2_LoF_ and high T2_WT_ scores (*p* < 0.05). Hypomorphic variants are significantly different from both controls (*p* < 0.05, Figure 2H; Table S2), suggesting transcriptional changes that are not simply an intermediate between LoF and WT. This is supported by differential expression analysis, where 48 of 141 (or 107 of 232) differentially expressed genes between hypomorphic versus WT control (or LoF) were not differential between the two controls, suggesting gain of new activity (Figure S6). Furthermore, variant fitness positively correlated with T2_WT_ scores (*r* = 0.85, *p* = 1.2e–32) and negatively with T2_LoF_ (*r* = −0.77, *p* = 8.4e–24), demonstrating that LoF-like variants result in increased fitness and larger cell numbers (Figures 2I and S4C; Table S2).

### Gene expression programs distinguish RUNX1 variants

**Figure 3.**
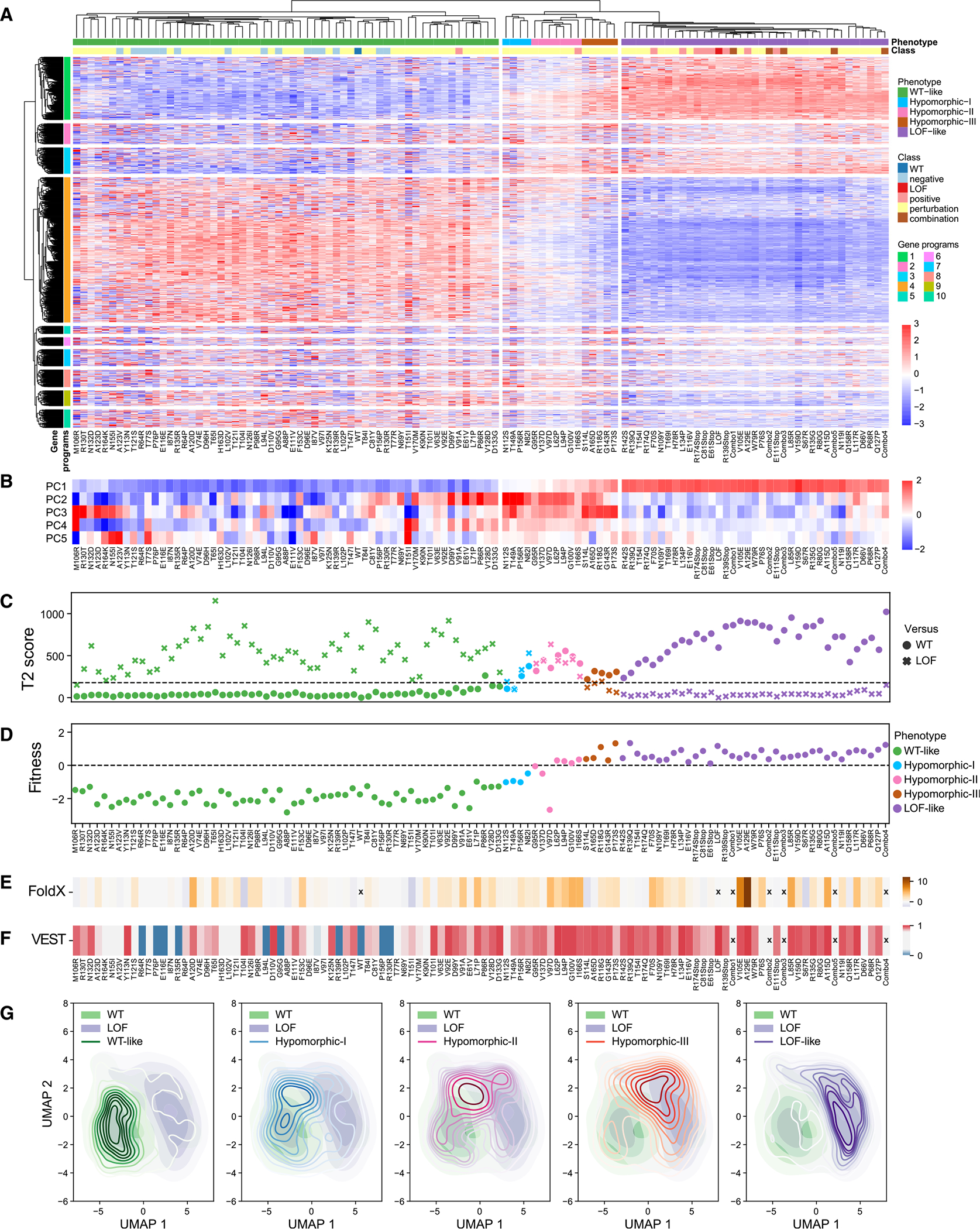
Mapping phenotypic consequences of RUNX1 variants with transcriptomic analysis (A) Hierarchical clustering of variants (columns: 5 clusters) by mean expression profiles of top 2,000 variable genes (rows: 10 clusters). Variant dendrogram leaves are ordered by increasing T2_WT_ scores. Gene expression values are *Z* scored. (B) Top 5 PCs of variants. Rows are scaled to have a mean of zero and unit variance. (C) Variant T2 scores when compared to the WT (circle) or LoF (cross) control, colored by phenotypes. Dotted line equals 178.79, median of T2_WT_ scores for all WT-like and LoF-like variants. (D) Variant mean fitness scores. (E and F) Variant (E) FoldX and (F) VEST scores. Variants that could not be scored (WT and LoF controls, or combination mutations) are grayed out and marked with an X. (G) Kernel density estimates comparing UMAP embedding of single cells belonging to each assigned phenotype (density lines) and to cells overexpressing the WT (green shade) or LoF controls (purple shade).

Hierarchical clustering of RUNX1 variants once again separated WT-like, LoF-like, and hypomorphic variants (Figure 3A). Although variants clustering with LoF control would most likely destabilize RUNX1 or interfere with its DNA binding, we did not want to assume that all hypomorphic variants would have the same effect on RUNX1 activity. Therefore, hypomorphic variants were further partitioned into three sub-groups based on the dendrogram (hypomorphic-I, -II, and -III), demonstrating a progression of expression changes (Figure 3A). The majority of variance in expression fell along the WT-like-to-LoF-like axis (PC1: 31.6%) (Figure 3B), correlating with T2_WT_ (*r* = 0.90, *p* < 2.2e–16), and fitness scores (*r* = 0.93, *p* < 2.2e–16); while PCs 2–4 (3.4%, 3.1%, and 2.6%, respectively) pointed to transcriptomic effects that are more specific to hypomorphic variants. In particular, hypomorphic-II variants displayed larger T2_WT_ and T2_LoF_ scores (Figure 3C), unlike the fitness progression (Figure 3D).

LoF-like variants produced higher FoldX and VEST scores (Figures 3E and 3F). Kernel density estimates of single-cell uniform manifold approximation and projection (UMAP) embeddings for variant groups demonstrated that the majority of cells belonging to each assigned phenotype occupy discrete regions in UMAP, although hypomorphic distributions also harbor cells that overlap with regions dominated by WT-like and LoF-like cells (Figure 3G). This could be due to small differences in the expression of the mutant construct, variability in knockdown of endogenous RUNX1 expression, stochasticity in the measurement of gene expression, or even variable penetrance at the cellular level due to buffering built into cellular systems, such as stress response pathways.

We identified gene clusters with similar expression patterns across variant groups by (1) hierarchical clustering and (2) non-negative matrix factorization (NMF) (CoGAPS^72^) of the top 2,000 variable genes. Functional enrichment analysis of 10 gene programs by hierarchical clustering (Figures 3A and S7; Tables S4 and S5) and 7 transcriptional patterns by NMF (Figure S8; Table S6) showed high concordance with some orthogonal results. WT-like variants displayed immune system and cell-cycle-related functions (patterns 4 and 1), positive regulation of T cell lineage commitment, proliferation, and activation specifically (program 4), consistent with the role of RUNX1 in hematopoietic lineage commitment and differentiation.^20,44,69,73,74^ LoF-like variants upregulated heme biosynthesis (program 1 and pattern 3), angiogenesis (program 2), and extracellular matrix regulation (program 3), which is consistent with a shift toward erythroid differentiation^49,50^ and hematological malignancies,^73,75^ with loss of RUNX1 activity. They also displayed enrichment for stress-response genes (patterns 6 and 7)-endoplasmic reticulum stress specifically-possibly suggesting that destabilizing RUNX1 mutations could trigger an unfolded protein response. Hypomorphic variants showed a higher expression of genes associated with tau protein kinase activity (program 9), suggested to link nerve growth factor (NGF) to activation of mitogen-activated protein kinase signaling,^76^ but lower expression for neuronal plasticity (program 7), suggesting effects on the less-known functions of RUNX1.^77–79^ They were also enriched for G1/S cell cycle and senescence (pattern 2), which could indicate G1 phase arrest for some hypomorphic variants, and interleukin (IL) and cytokine signaling (pattern 5), suggesting a shift toward an inflammatory state for some of the cells.^41,80–83^

### LoF-like variants significantly target DNA binding, while hypomorphic variants favor protein interfaces

**Figure 4.**
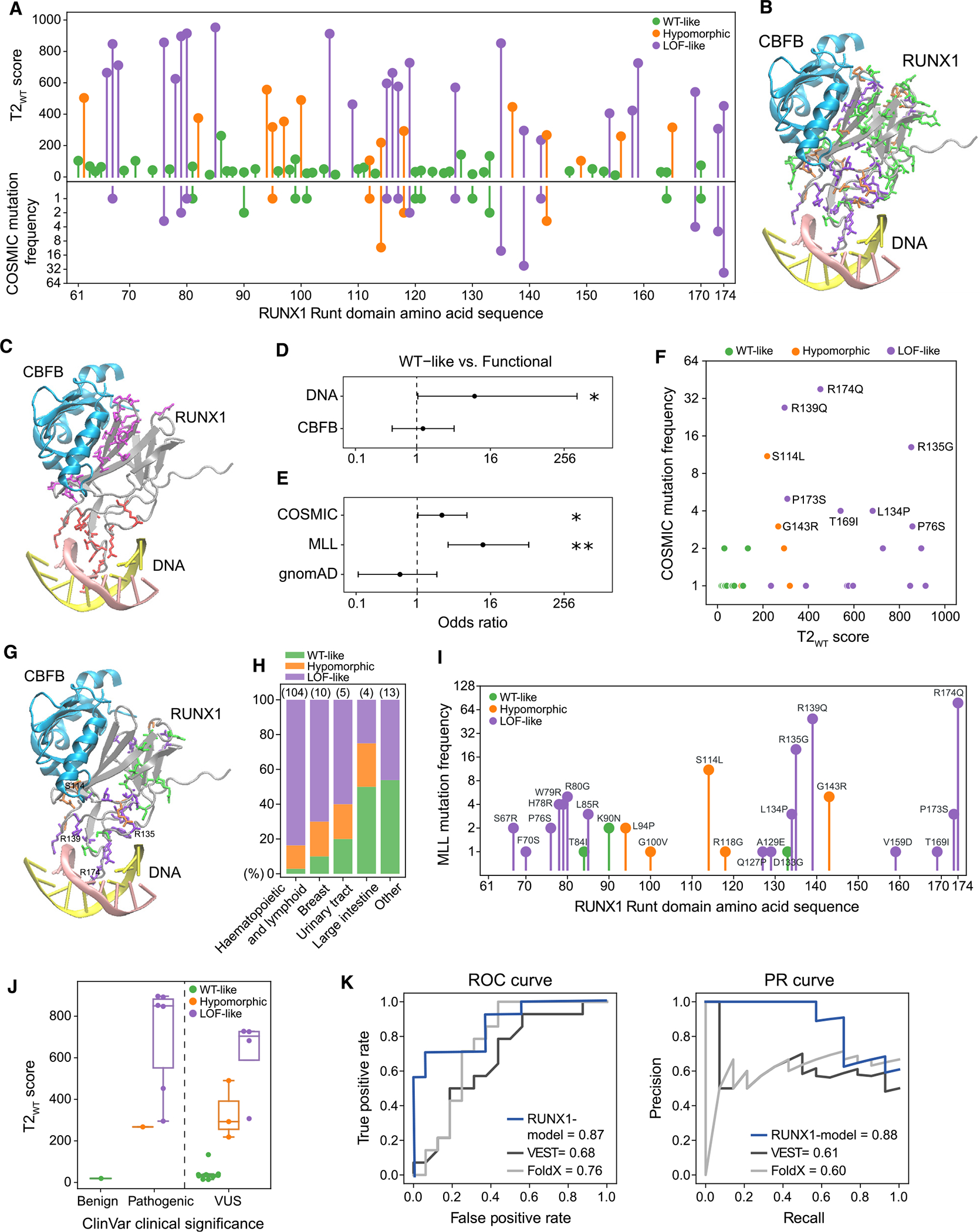
Mapping oncogenic variants onto the RUNX1 regulatory landscape (A) Sequence-based phenotypic profiling of 79 RUNX1 perturbation variants. Top: variant T2_WT_ scores; bottom: mutation frequency in cancer (COSMIC) (log2 scaled). (B, C, and G) Structure-based phenotypic profiling of RUNX1 perturbation variants. The 3D crystal structure of transcription factor CBF, consisting of RUNX1 Runt domain (gray) and CBFB (blue), interacting with DNA (yellow and pink strands) (PDB: 1h9d). Amino acid residues corresponding to (B) all 79 perturbation variants and (C) variants targeting DNA (red) or CBFB interaction (purple), or (G) observed in cancer (COSMIC), colored by phenotypic designations. The 4 most frequent mutations are annotated. (D and E) ORs with 95% confidence intervals. Enrichment of WT-like vs. functional (LoF-like or hypomorphic) impact variants (D) for DNA- or CBFB-binding residues, or (E) in cancer vs. non-cancer genome databases. OR >1 indicates enrichment for functional variants, while OR <1 means depletion (*p < 0.05, **p < 0.001). (F) T2_WT_ scores vs. mutation frequency (log2 scaled) of library variants present in cancer (COSMIC). (H) Percent distribution of variant phenotypic annotations across tumors observed in different primary tissues. Sample size for each tissue is displayed on top. The 4 most frequent tissue types are shown. (I) Frequency of mutations in MLL overlapping variants in the RUNX1 library (log2 scaled). (J) T2_WT_ scores of germline variants, grouped according to clinical significance and colored by variant phenotypic annotations. (K) Performance of “RUNX1-model” classifier vs. VEST and FoldX, summarized by the AUROC and AUPR scores.

We noted local clustering of variants with similar functional effect along the RUNX1 Runt domain amino acid sequence (Figure 4A), and the three-dimensional (3D) structure, especially in the DNA-binding region (Figure 4B). We identified 11 amino acid residues that directly contact DNA (STAR Methods), among which 8 are perturbed in our mutation library (R80G, R135G, R139Q, R142S, G143R, T169I, V170M, and R174Q) (Figure 4C), and these are significantly enriched for functional (LoF-like or hypomorphic) vs. WT-like impact (OR = 8.82, *p* = 0.025; Figure 4D), consistent with reports that mutations to DNA-contact residues severely impair RUNX1 function.^24,59,84,85^ Transcriptome-based (T2) scores recapitulated experimental findings (Table S7); six mutated positions scoring as LoF-like disrupted DNA binding in alanine-scanning mutagenesis assays,^23,25^ while the position with WT-like impact (V170M) did not, and the position with a hypomorphic mutation (G143R) perturbed the Runt domain fold instead.^23^

In comparison, for 19 residues mediating the CBFB interaction (Figure 4C; STAR Methods), enrichment for functional effects is weaker; only 10 mutations are functional, while 9 are WT-like (OR = 1.27, *p* = 0.79; Figure 4D). Seven involve residues experimentally shown to not perturb CBFB binding^23,24,86^ (Table S7), which is in line with our WT-like designations. Moreover, our assay identified the N109Y mutation of residue 109, a hotspot for CBFB heterodimerization,^86^ as LoF-like. Notably, mutations interrupting CBFB binding but not DNA have been suggested to produce hypomorphic alleles,^84^ and one such case was identified by our assay (hypomorphic T149A). In fact, of 14 hypomorphic variants found, 4 occurred at the CBFB interaction interface, with only 1 at the DNA. Overall, transcriptome-based scores reflect the difference in sensitivity to perturbations of protein- versus DNA-binding interfaces.^84^

### Comparing coding variant Perturb-seq with recurrence in cancer

In principle, mutations that improve fitness would be selectively more advantageous for tumor cells and show higher recurrence across patients. While we observed significant overrepresentation of functional mutations in cancer (COSMIC^64^) (OR = 3.05, *p* = 0.022; Figure 4E), frequency only weakly correlated with fitness (*r* = 0.33, *p* = 1.2e–3) and not with T2_WT_ scores (*r* = 0.15, *p* = 0.14; Figure 4F). This suggests that while recurrence is a strong indicator of functional impact, it does not distinguish differences in the magnitude of effect well, highlighting the importance of transcriptomic studies to assess variant impact. Notably, the top three most frequent mutations (R174Q, R139Q, and R135G) target DNA-contact residues and display LoF-like impact, while the fourth (S114L) targets CBFB heterodimerization and is hypomorphic (Figure 4G). Furthermore, the top hotspot mutation (R174Q) is known to contribute the greatest energy to DNA binding.^23^ Of the RUNX1 mutations shared between our assay and COSMIC, the majority occurred in hematopoietic malignancies (*n* = 104), followed by breast cancer (*n* = 10), urinary tract (*n* = 5), and large intestine (*n* = 4), where approximately 79.6% are LoF-like and 14.6% are hypomorphic (Figure 4H).

We revisited RUNX1 mutations in a larger set of hematopoietic malignancies from the Munich Leukemia Laboratory (MLL) (STAR Methods), which contains 717 tumors with somatic missense mutations in the Runt domain, 201 tumors of which capture 24 unique variants present in our library (Figure 4I). We again observed a bias for functional mutations (OR = 11.03, *p* = 7.6e–5; Figure 4E), but even higher than in COSMIC, consistent with the significance of RUNX1 in AML. The same mutations had the highest frequencies (R174Q, R139Q, R135G, and S114L)^87^ (Table S2). In contrast, we see a trend toward the depletion of functional variants in non-cancer genomes from the gnomAD database^88^: 4 functional vs. 8 WT-like (OR = 0.52, *p* = 0.37; Figure 4E), and 3 of the functional variants were annotated as pathogenic in ClinVar.^87^

### Transcriptome-based phenotyping informs variants of uncertain significance

Germline RUNX1 variants are associated with familial platelet disorder, characterized by an increased risk of developing myeloid malignancies.^57,89,90^ However, information about the consequences of many are lacking, leading to a variant of uncertain significance (VUS) designation, which presents a challenge for clinical interpretation.^91^ We obtained 148 unique RUNX1 Runt domain missense mutations from ClinVar,^87^ 24 of which overlap with our library, with 1 benign, 7 pathogenic, and 16 VUS germline significance annotations (STAR Methods). Our transcriptome-based profiling recapitulated the 8 benign/pathogenic ClinVar variants with 100% accuracy (Figure 4J; Table S2), suggesting that transcriptomic labels can be used to reevaluate VUSs. Of the 16 present in our library, we identified 9 as WT-like, 3 as hypomorphic, and 4 as LoF-like, providing new evidence in support of reclassifying these VUSs (Figure 4J).

### Using transcriptome-based labels for variant effect prediction

COSMIC, MLL, and ClinVar datasets encompass missense mutations that are not in our library, either different amino acid substitutions of included positions or substitutions of others. We reasoned that our library could serve as training data to predict their functional effects. The 79 perturbation variants in our library (41 WT-like, 24 LoF-like, 14 hypomorphic) were divided into a training set and a test set at random with balanced ratios of functional vs. WT-like. We annotated each variant with 85 features describing substitution effects on amino acid biophysical properties (SNVBox^92^) and trained a random forest classifier on the training set to predict functional vs. WT-like variant labels (STAR Methods). Our RUNX1-specific model scored 0.87 area under the receiver operating characteristic curve (AUROC) and 0.88 area under the precision recall curve (AUPR) on the test set, outperforming sequence-based variant effect and protein stability predictions from VEST and FoldX (Figure 4K).

Encouraged by these results, we trained a classifier on all 79 perturbation variants and evaluated performance on the positive and negative control missense variants in our library (*n* = 12), obtaining 0.81 AUROC and 0.84 AUPR scores. We then predicted transcriptomic effect labels of all the remaining possible missense mutations of RUNX1 (*n* = 2,582) (Table S8), resulting in 302 functional and 355 WT-like predictions for Runt domain mutations not contained in our library. For mutations observed in cancer (Figure S9A), predictions were biased toward being functional (COSMIC: 101 functional vs. 52 WT-like, OR = 2.92, *p* = 1.9e–8; MLL: 109 functional vs. 36 WT-like, OR = 4.99, *p* = 1e–15); whereas gnomAD database mutations were biased toward WT-like (27 functional vs. 50 WT-like, OR = 0.59, *p* = 0.051; Figure S9B). We further assessed the performance of our classifier on a high-confidence subset of 110 pathogenic vs. 74 neutral Runt domain variants assembled from COSMIC, MLL, ClinVar, and gnomAD databases (STAR Methods), achieving 0.79 AUROC and 0.82 AUPR scores (Figure S9C). For 21 unique germline variants with benign/pathogenic clinical annotations (ClinVar), we achieved 0.81 accuracy (only 4 false negatives), giving confidence to our model’s predictions on 103 remaining VUSs (45 functional vs. 58 WT-like; Figure S9D). We used a conservative score threshold (0.5) to assign functional vs. WT-like predictions, which provided reasonable separation in both cancer and germline cases, but relaxing the threshold within the 0.4–0.5 range could increase accuracy (Figures S9D and S9E).

### Hypomorphic variant impact on the RUNX1 regulon

**Figure 5.**
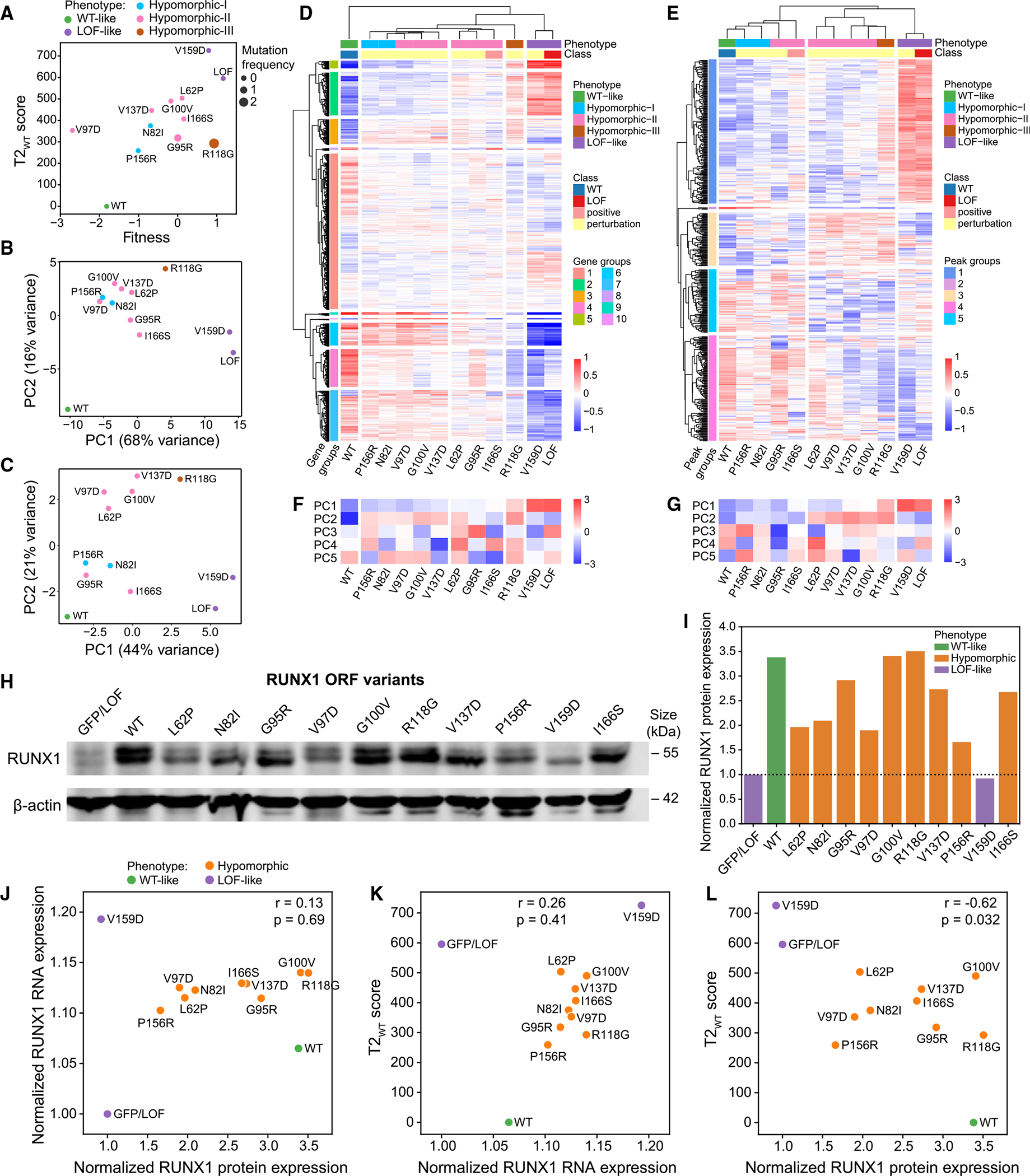
Bulk RNA-seq, ATAC-seq, and western blot analysis of 12 validation variants (A) Overview of validation variants: T2_WT_ and fitness scores (from scRNA-seq analysis), and mutation frequency in cancer (COSMIC). (B and C) PCA of variants, in bulk (B) RNA-seq (using top 2,000 variable genes of scRNA-seq analysis) or (C) ATAC-seq using top 500 variable peaks. Gene expression and DNA accessibility are averaged across replicates. (D and E) Unsupervised hierarchical clustering of variants (columns) and (D) genes (rows) in bulk RNA, or (E) peaks (rows) in ATAC-seq. Gene expression and DNA accessibility are averaged across replicates and mean centered. Leaves of variant dendrograms are ordered by increasing T2_WT_ scores. (F and G) Top 5 PCs of variants based on mean (F) gene expression or (G) DNA accessibility across replicates. Rows are *Z* scored. (H) Western blot quantifying RUNX1 protein levels in K562 cells transduced with a validation variant (columns), with β-actin acting as a loading control. Here, endogenous RUNX1 was not knocked down; therefore, the GFP/LoF construct represents endogenous RUNX1 expression. (I) Variant protein expression normalized to β-actin control and to endogenous RUNX1 levels captured by the GFP/LoF construct (dashed line). (J) Variant distribution of normalized RUNX1 RNA vs. protein expression (Pearson’s r = 0.13, *p* = 0.69). (K and L) Variant distribution of T2_WT_ scores vs. normalized RUNX1 (K) RNA (Pearson’s r = 0.26, *p* = 0.41), or (L) protein expression (Pearson’s r = −0.62, *p* = 0.032).

To validate hypomorphic effect variants and investigate their impact on the RUNX1 regulon, we performed bulk RNA sequencing (RNA-seq) and assay for transposase-accessible chromatin with sequencing (ATAC-seq). We selected 12 variants to study, including WT and LoF controls, 9 hypomorphic variants that showed largest deviations from both controls, and an LoF-like variant (V159D) predicted to target RUNX1-CBFB binding, to further investigate effects of its interruption (Figure 5A; Table 1). Bulk screens were performed for each variant separately in iRUNX1-KD K562 cells grown in dox to induce repression of endogenous RUNX1 (dox condition), and hygromycin to select for transduced cells. Each variant contained 3 biological replicates with more than 1 million cells. At day 7 post-transduction, cells were split into two groups: ~1 million and 100,000 cells to be sequenced to a depth of 30 or 75 million reads per sample for bulk RNA- and ATAC-seq (Figure 1D).

After alignment, QC filtering, normalization, removal of replicate-specific batch effects, and averaging across replicates, we obtained an 18,646 genes by samples expression matrix and an 82,870 peaks by samples count matrix (STAR Methods). Principal-component analysis (PCA) of variants on bulk gene expression and ATAC-seq peaks showed similar trends, with PC1s reflecting progression of phenotypic effects, from WT-like to hypomorphic-I, -II, -III, to LoF-like, while PC2s distinguished hypomorphic variants from both WT and LoF (Figures 5B and 5C). Unsupervised hierarchical clustering of variants on bulk gene expression (Figure 5D) reproduced the earlier single-cell (sc)RNA-seq-based clustering (Figure 2A), supporting the idea that single-cell transcriptomic analysis can reliably identify hypomorphic variants. Unsupervised hierarchical clustering of variants on ATAC-seq peaks produced a similar ordering (Figure 5E), suggesting that this peak set contains information relevant to variant-specific expression effects. PC3–5 captured more subtle distinctions of hypomorphic variants in both cases (Figures 5F and 5G). Analyzing replicates separately yielded similar results (Figure S10).

Variant protein expression was verified by western blot (Figure 5H), which revealed that LoF-like variant V159D resulted in protein loss, possibly due to unstable protein, while hypomorphic variants showed variable protein levels ranging from 1.6–3.5 times the endogenous RUNX1 expression (represented by the GFP/LoF control) (Figure 5I). Variant RNA expression showed little variation between hypomorphic variants and did not correlate with protein expression (*r* = 0.13, *p* = 0.69; Figure 5J), or T2_WT_ scores (*r* = 0.26, *p* = 0.41; Figure 5K). When WT and LoF-like controls were included, T2_WT_ scores negatively correlated with variant protein expression (Figure 5L), but showed no correlation for hypomorphic variants only (*r* = 0.13, *p* = 0.74). Thus, hypomorphic variant effects are not explained solely by variation in variant RUNX1 levels, supporting that functional consequences are likely due to the altered regulation of RUNX1 target genes.

**Figure 6.**
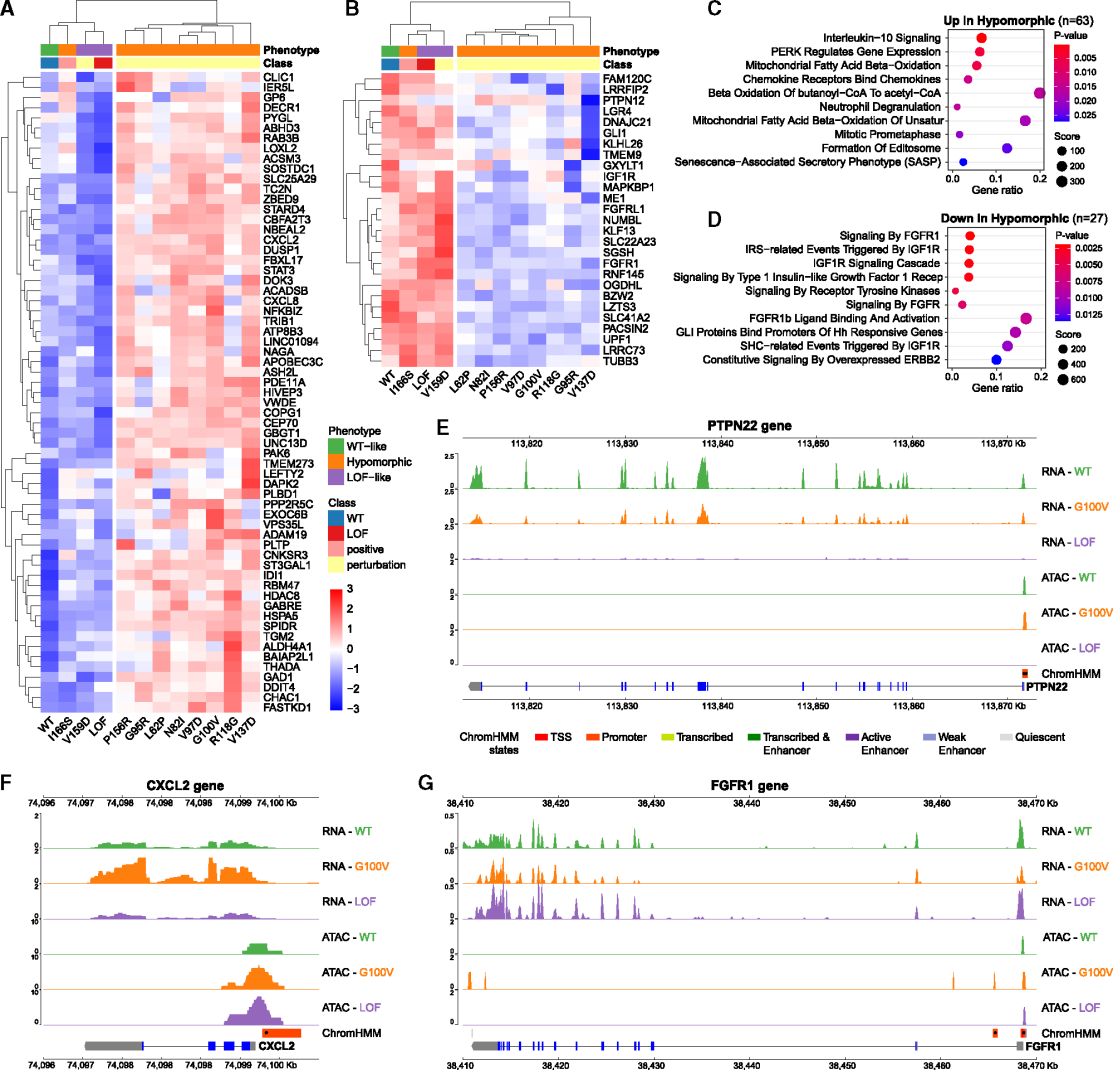
Regulatory consequences of hypomorphic RUNX1 variants (A and B) Hierarchical clustering of variants (columns) in bulk RNA-seq for genes (rows) with ATAC peaks and RUNX1 motifs in their promoters that are (A) upregulated (*n* = 63) or (B) downregulated (*n* = 27) in at least 1 hypomorphic variant against both WT and LoF controls. Gene expression is averaged across replicates and *Z* scored. (C and D) Overrepresentation of Reactome pathways for genes in (A) and (B), respectively. Top 10 pathways, ordered by *p* values, are displayed. (E–G) RNA-seq and ATAC-seq tracks of 3 example genes demonstrating distinct hypomorphic effects: (E) PTPN22 shows partial LoF, while (F) CXCL2 and (G) FGFR1 display gain of function or LoF against both WT and LoF. Tracks are displayed for WT and LoF controls along with the hypomorphic G100V variant. ATAC peaks are annotated with ChromHMM states, with asterisks indicating RUNX motifs. Gene exons and UTRs are represented with blue and gray bands.

To investigate whether hypomorphic variants altered DNA accessibility at regulatory elements with RUNX1-binding motifs near differentially expressed genes, we studied 202 and 89 genes that were significantly up- or downregulated, respectively, in at least 1 hypomorphic variant relative to both WT and LoF controls (false discovery rate < 0.05; STAR Methods). Of these, 63 and 27 (Figures 6A and 6B), respectively, had ATAC peaks in their promoter regions ([−1 kb, +100 bp] of transcription start sites) with RUNX1-binding motifs, suggesting direct regulation by RUNX1. Analyzing these genes for functional enrichment (Table S9) suggested hypomorphic variants may upregulate IL-10 signaling and PERK-regulated gene expression (Figure 6C) but downregulate fibroblast growth factor receptor 1 (FGFR1) and IGF1R-regulated signaling (Figure 6D). To further evaluate specific genes, we visualized RNA and ATAC profiles of cells carrying hypomorphic variants relative to cells with WT and LoF controls. As an example of a gene on a continuum from WT to LoF, PTPN22 shows an intermediate effect of gene expression for the hypomorphic cancer variant G100V (Figure 6E). In contrast, CXCL2, involved in IL-10 signaling, and FGFR1, main driver of FGFR1 signaling, demonstrated potential gain-of-function or LoF activity relative to both WT and LoF controls (Figures 6F and 6G). RUNX1 mutations are reportedly more frequent in the context of FGFR1 translocations, which have been linked to more aggressive disease.^93^ For FGFR1, we observed multiple ATAC peaks in the hypomorphic case not observed in WT or LoF, possibly suggesting effects of the variant on targeting RUNX1 to sites with inhibitory activity on gene expression.

Several additional differentially up- or downregulated genes specific to hypomorphic variants had RUNX1 motifs in nearby enhancers (Figures S11A and S11B). Some of them, including STAT3 and MAPKBP1, had nearby ATAC peaks with RUNX1 motifs both at their promoter and a nearby enhancer, making it difficult to discern which element contributed to the altered expression. Upregulated genes again showed enrichment for IL-10 signaling, but also in NGF-stimulated transcription and NTRK1-regulated signaling, while downregulated genes were enriched in NFAT activation and BCR signaling (Figures S11C and S11D; Table S10). Available chromatin conformation capture data for K562 cells supported chromatin looping between enhancers containing RUNX1 motifs and up- or downregulated genes. For example, we observed loops linking enhancers to the increased expression of CD24 for V137D, and to decreased expression of RPP25 for cancer variant R118G, relative to both WT and LoF controls (Figures S11E and S11F). In both cases, ATAC profiling of the hypomorphic variant suggested involvement of enhancers with RUNX1 motifs, whereas ATAC peaks were not observed for WT or LoF, illustrating that hypomorphic variants can perturb both transcription enhancing and inhibitory functions of RUNX1 on gene regulation. However, it is important to note that secondary effects from downstream RUNX1 regulon genes may also play a mechanistic role in governing this differential regulation.

## DISCUSSION

While evidence shows that different mutations affecting the same cancer gene can lead to differences in disease severity^6,7,94^ or drug sensitivity,^5,95,96^ the potential for pleiotropy to drive heterogeneous tumor cell phenotypes remains poorly understood. We used information about physical contacts between proteins to guide the design of a library of amino acid substitutions. We selected RUNX1 for its well-studied role in cancer and as a master regulator of hematopoiesis, reasoning that mutations here could produce large detectable differences in gene expression. We designed mutations based on *in silico* prediction of their potential to disrupt RUNX1 interactions with co-factors and profiled their transcriptional consequences at the single-cell level with SEUSS, which revealed three functional groups. Most mutations had effects similar to LoF or WT, except for 15 that generated transcriptional profiles different from both extremes (hypomorphic). Comparison with other experimental RUNX1 mutation studies showed that transcriptome-based profiles recapitulate differences detected through affinity-based methods and alanine-scanning mutagenesis.

This study makes two updates to our original SEUSS vector design to improve signal in screens: (1) to eliminate issues with barcode shuffling, the variant and its barcode are positioned in direct proximity; and (2) to ensure minimal modification to the ORF, variants are positioned downstream of the 2A peptide so that only a proline gets appended to the protein sequence N terminus. Simultaneous CRISPRi knockdown of endogenous RUNX1 further boosted effect sizes. Notably, the differences in transcriptional profiles of hypomorphic variants at the single-cell level reproduced robustly in bulk RNA, supporting SEUSS as a viable strategy for investigating the relatively subtle differences in gene expression that we observed for missense variants.

The role of RUNX1 as an oncogene vs. tumor suppressor is still not entirely clear and may depend on the type of malignancy, as well as other mutations present. In humans and *in vivo* model systems, loss of RUNX1 leads to increased susceptibility to AML; point mutations are associated with shorter time to progression from MDS to AML and worse prognosis in AML and CML.^97,98^ In contrast, for RUNX1 translocations, a survival dependency on WT RUNX1 has been reported.^99^

In this single-cell experiment with K562 background, the majority of mutations resulted in WT-like effects, although initially predicted to be functional by *in silico* pathogenicity prediction, emphasizing the value of this transcriptomic variant assay. LoF mutations, especially those targeting DNA binding, displayed higher fitness with larger cell counts, suggesting that RUNX1 acts as a tumor suppressor in this setting of CML with blast crisis, and its loss provides a selective growth advantage, although it is important to note that K562 cells represent disease that developed on a WT RUNX1 background. Notably, the five constructs carrying multiple mutations generated even more extreme effects than the LoF control that replaced RUNX1 with GFP. Furthermore, hypomorphic variants tended to perturb protein interactions vs. DNA, consistent with reports that RUNX family mutations at DNA-contact residues severely impair function, resulting in hematopoietic disease. In contrast, those interrupting CBFB binding generate hypomorphic alleles,^84^ resulting in a skeletal disorder, cleidocranial dysplasia,^85^ suggesting that our transcriptomic profiling shows sensitivity to distinguish pleiotropic effects.

Hypomorphic variants showed subtle transcriptomic differences with LoF variants, which was confirmed by bulk RNA-seq. Although small, these differences affected important signaling pathways (NGF-stimulated transcription, IL-10 signaling, PERK activity) and cancer genes (CBFA2T3, ETV4, FGFR1, GLI1, SGK1, and STAT3). Cells harboring hypomorphic variants downregulated a transcriptional program associated with neuronal plasticity while overexpressing genes associated with the response to NGF. Previous studies implicate neurotrophic signaling as a promoter of malignant cell growth and survival across a variety of tumor types.^100^ Hypomorphic variants displayed lower expression of FGFR1, perhaps indicating a different pattern of reliance on growth factors with links to neuronal plasticity.^101^ Certain immune functions were also altered; IL-10 signaling molecules CXCL2 and CXCL8, implicated in neutrophil recruitment,^102^ were higher in the hypomorphic case, whereas NFATC1, a mediator of T cell activity, was downregulated. Physiological implications of these small differences in tumor microenvironment are unclear, although increased neutrophil-to-lymphocyte ratio has been associated with poor prognosis.^103^

A number of mutations in our library were recurrently observed across multiple tumors, a phenomenon usually associated with oncogenes^1^; however, the most recurrent events still clustered with the LoF control. Several hypomorphic variants were also seen in multiple tumors, although at a lower level of recurrence. In single-cell plots, individual mutations were difficult to distinguish without first mapping to densities, and even then, some cells coincided with WT or LoF regions. Further investigation is needed to understand whether this reflects stochastic differences in construct expression or knockdown of endogenous RUNX1, cell-to-cell differences in read coverage, or bona fide variable penetrance of the variant effect on the phenotype of individual cells.

### Limitations of the study

This study was performed in K562 CML cells, which express WT RUNX1 and have been extensively characterized by the ENCODE project. Although altered RUNX1 is most commonly associated with AML, there are reports of frequent RUNX1 mutations in blast crisis CML,^104,105^ and we showed that functional variants in K562 are significantly overrepresented in AML (MLL cohort). Future studies in additional cell lines are needed to determine how well the effects generalize to other leukemias or non-hematopoietic tumor types. Our screen uses overexpression constructs to introduce single-nucleotide mutations, producing RUNX1 protein levels 1.6–3.5 times higher than endogenous levels. Other approaches include base editing,^106,107^ which generates specific mutations at the endogenous locus that would more closely recapitulate endogenous levels, but is subject to other limitations, including incomplete editing, that not all sites in the genome can be targeted, and that not all base pair changes can be generated. In addition, we focused only on the Runt domain, whereas other domains are also important for RUNX1 cofactor interactions. Thus, we may not have fully captured the space of possible phenotypes that can be produced by single amino acid substitutions in RUNX1. Furthermore, our epigenetic profiling was limited to DNA accessibility, whereas chromatin immunoprecipitation sequencing would more directly reveal mutation-associated changes to RUNX1 localization. Additionally, direct assays of protein binding could further confirm that hypomorphic variant impact is caused by altered targeting of RUNX1 to DNA sites, rather than protein stability loss, as suggested by the lack of correlation between T2 scores and protein expression. These questions will be the topics of future studies to better understand the role that Perturb-seq can play in providing exploitable mechanistic insights in cancer.

## STAR★METHODS

### RESOURCE AVAILABILITY

#### Lead contact

Further information and requests for resources and reagents should be directed to and will be fulfilled by the lead contact, Hannah Carter (hkcarter@health.ucsd.edu).

#### Materials availability

This study did not generate new unique reagents. The constructs introduced into cell lines are detailed in the key resources table.

#### Data and code availability

- All sequencing datasets are available in the NCBI BioProject database under accession number PRJNA1121229, specifically scRNA-seq: PRJNA1033389, bulk RNA-seq: PRJNA1121326 and bulk ATAC-seq: PRJNA1121327.
- All original code is available under an MIT license via Github repository https://github.com/cartercompbio/RUNX1_SEUSS.
- Any additional information required to reanalyze the data reported in this work is available from the lead contact upon request.

### EXPERIMENTAL MODEL AND STUDY PARTICIPANT DETAILS

#### Cell lines

HEK293T and K-562 cells were purchased from ATCC (ATCC #CRL-3216 & ATCC #CCL-243, respectively). HEK293T were grown in DMEM media (ThermoFisher Scientific #10566016) supplemented with 10% FBS (Gibco #A52568) and 1% antibiotic-antimycotic (Gibco #15240-062), and cultured at 37°C with 5% CO_2_. K-562 cells were grown in RPMI 1640 media (Gibco ##11875-093) supplemented with 10% FBS and 1% antibiotic-antimycotic and cultured at 37°C with 5% CO_2_.

### METHOD DETAILS

#### RUNX1 reference

Variant residue positions were defined based on the RUNX1B isoform of the RUNX1 gene (ENSG00000159216), corresponding to the Q01196-1 isoform protein (453 amino acids) described as the canonical sequence in the UniProt database,^61^ encoded by transcript ENST00000344691 (7274 base pairs)^127^ and NM_001001890. The Runt domain is ~128 amino acids long, corresponding to amino acid positions 50–177 in the RUNX1 protein.^85,128^

#### Protein 3D structure analysis

We obtained 61 experimentally verified undirected protein interactions of RUNX1 with a confidence score higher than 0.4 from STRING v9.1.^126^ Experimental 3D co-crystal protein structures for RUNX1-CBFB interaction (PDB: 1ljm, 1e50, 1h9d) were obtained from the Protein DataBank (PDB),^129^ and used to predict amino acid residues of RUNX1 in direct physical contact with CBFB as described in our previous work.^130^ The remaining interactions did not have co-crystal structures. Instead, we used *in silico* template-based protein docking on single protein structures with PRISM^60^ to identify contact residues. PRISM returned predictions for 33 RUNX1 interaction partners (Figure 1B).

Amino acid residues of RUNX1 involved in DNA binding (PDB: 1h9d) were determined using the distance between two non-hydrogen atoms of amino acids and nucleotides, one from the protein and one from the DNA. If the distance was less than 3.5A, we designated them as interface residues,^17^ which identified R80, R135, R139, R142, G143, K167, T169, V170, D171, R174, R177 as DNA-contact residues. Amino acid residues were annotated as core, surface, or intermediate based on their relative solvent accessible surface areas as described in our previous work.^130^ VMD^131^ was used to visualize protein 3D structures (Figures 1A, 4B, 4C, and 4G).

#### Selection of variants for library construction

The ORF mutation library consists of 117 elements: 83 single amino acid substitutions at protein interaction interfaces in the RUNX1 Runt domain, 1 WT construct, 1 LOF construct, 17 negative, and 10 positive control mutations, and 5 combinations of two or more interface mutations (Figures 1D; Table S1). Variant effect prediction scores for all possible missense mutations targeting each residue were obtained from VEST^62^ and FoldX.^63^ Variant frequency in human tumors was determined from the COSMIC database^64^ (obtained on 11/7/2022, for transcript ENST00000344691), along with the primary tissue the tumor resides in (Figure 4H). For each residue, the most damaging amino acid substitution possible from a single base substitution (the highest VEST or FoldX scored mutation) was chosen to be included in the ORF mutation library, prioritizing cancer mutations where possible, to maximize the possibility of perturbing physical protein interactions. 30 of 83 mutations tested are cancer mutations.

The WT construct consists of WT RUNX1, while the LOF construct contains a green fluorescent protein (GFP) in place of RUNX1. 17 negative control mutations consist of 10 silent and 7 neutral (predicted based on VEST scores) mutations and are expected to be functionally indistinguishable from the WT construct. 10 positive controls consist of 5 truncating and 5 core mutations and are expected to have similar impact to the LOF construct, by resulting in a truncated or unstable protein. 5 perturbation mutation combinations consist of combinations of two, three or four perturbation mutations already in the library (Table S1).

#### Building of RUNX1 variant library

The gene overexpression vector was generated from a modified lentiviral vector (Addgene #120426). The vector was modified by removing both the mCherry transgene and the hygromycin resistance enzyme gene. The hygromycin resistance enzyme gene was then re-cloned to be immediately downstream of the EF1a promoter, followed by a P2A peptide motif and a NheI restriction site, which was used to clone in the library elements. A 12 base pair barcode sequence was then introduced downstream of the cloning site to identify variants during single-cell transcriptome sequencing (Table S11). To insert the barcode, the backbone was digested with NheI (New England BioLabs), and a pool of 12 base pair long barcodes with flanking sequences compatible with the NheI site was cloned using Gibson assembly. To clone the library elements, the expression vector was digested with NheI for 3 h at 37°C. The linearized vector was then purified using a QIAquick PCR Purification Kit (Qiagen).

DNA fragments coding for the library elements were ordered from Twist Bioscience as a site saturation variant library in an arrayed format as linear dsDNA. A fraction of each oligonucleotide encoding the corresponding variant was then combined, and the pool was amplified via PCR using KAPA-Hifi (Kapa Biosystems) in 50 μL reactions containing 10 ng of pooled template and 2.5 μL of primers RX1_01 and RX1_02 (10 mM), which include ~30 bp of DNA homologous to the overexpression vector to enable Gibson assembly cloning. A thermal cycler was used to heat the sample to 95°C for 3 min, then 16 cycles of 98°C for 20 s, 68°C for 15 s, and 72°C for 45 s, followed by a final 5 min extension at 72°C. The PCR products were then purified using Agencourt AMPure XP Beads (New England BioLabs) beads at a 0.8:1 bead:PCR reaction ratio. See Table S12 for primer sequences.

Gibson assembly was then used to clone the pooled library elements into the overexpression vector. For the reaction, 50 ng of the digested vector and 30 ng of the insert were mixed with 5 mL of Gibson Reaction Master Mix (New England BioLabs) in a reaction volume of 10 μL. The Gibson reactions were incubated at 50°C for 1 h and transformed via heat shock into 50 μL of One Shot Stbl3 chemically competent cells (Invitrogen). This was done by incubating the cells with the Gibson on ice for 30 min, followed by a 45 s heat shock at 42°C then 2 min on ice, then the addition of 250 μL of SOC media (Thermo Fisher Scientific). The cells were allowed to recover shaking at 37°C for 1 h and were then plated on LB-carbenicillin plates. Individual bacterial colonies were picked off of the plate and grown in LB-carbenicillin culture media shaking for 16 h at 37°C. After growth, plasmid DNA was isolated via a Qiagen Plasmid Mini Kit. Each colony was Sanger sequenced using the primer RX1_03 to identify the variant, then by the primer RX1_04 to capture the associated barcode. One overexpression vector was created for each variant, each with a single unique barcode associated. After ~30% of the library was cloned, the oligonucleotides for remaining elements were re-pooled and cloned using the above protocol, until the full library was assembled. To generate the combination mutations, the first mutation was created as described above. Subsequent mutations were generated with overlap extension PCR with primers containing the desired mutations.

#### Lentivirus production

Replication deficient lentiviral particles were produced in an arrayed format for each element of the library in HEK293FT cells (Invitrogen) via transient transfection. The HEK293FT cells were grown in DMEM media (Gibco) supplemented with 10% FBS (Gibco) and 1% antibiotic-antimycotic (Thermo Fisher Scientific). One day prior to transfection, HEK293FT were plated in 12 well plates at ~35% confluency, giving one well per element of the library. The day of transfection, the culture medium was removed and replaced with fresh DMEM plus 10% FBS. Meanwhile, the transfection mix was prepared by mixing 125 μL of Optimem reduced serum media (Life Technologies) with 1.5 μL of lipofectamine 2000 (Life Technologies), 125 ng of pMD2.G plasmid (Addgene #12259), 500 ng of pCMV delta R8.2 plasmid (Addgene #12263), and 375 ng of each plasmid overexpression vector for each library element. The transfection mix was incubated for 30 min, then added dropwise to the HEK293FT cells. The viral particles in the supernatant were harvested at 48 and 72 h post transfection, and the virus for each library element were pooled and filtered with a 0.45 mm filter (Steriflip, Millipore), then concentrated to 1.5 mL using Amicon Ultra-15 centrifugal filters with a cutoff 100,000 NMWL (Millipore). The virus was then mixed, aliquoted and frozen at −80°C. For the validation screen, the transfection was performed in 15 cm dishes, one for each of the selected validation mutations, and frozen separately.

#### Generation of clonal inducible RUNX1 repression cell line

To repress the endogenous RUNX1, the repression vector was generated from a PiggyBac inducible dCas9 construct (Addgene #63800). The vector was modified by removing the inducible transgene, and the sequence for the KRAB-dCas9 fusion (Addgene #60954) followed by a P2A sequence then GFP was inserted in its place. The vector was then modified through the insertion of a U6 promoter followed by SaII and AflII cloning sites for insertion of guide RNA sequences, then a guide RNA scaffold. Guides for CRISPRi targeting RUNX1 were chosen from the Dolcetto library set A^132^ and ordered via oligonucleotide from IDT. The guides were then cloned into the repression vector after digestion with SaII (New England BioLabs) and AflII (New England BioLabs).

K562 cells (ATCC) were cultured in RPMI 1640 media (Gibco) supplemented with 10% FBS and 1% antibiotic-antimycotic. One day prior to electroporation, the K562 cells were maintained at a concentration of 1 million cells per mL. The day of the electroporation, the cells were spun down and resuspended at a concentration of 10 million cells per mL. A total of 2 μg of DNA was added to 100 μL of cells containing a 1:2.5 M ratio of the all-in-one RUNX1 targeting repression vector to the PiggyBac transposase vector (Transposagen). The DNA was then electroporated into the K562 cells using the Ingenio Electroporation Kit (Mirus Bio) and a 4D Nucleofector (Lonza) per the manufacturer’s protocol. The cells were recovered for 3 days, then selected for those that received integration by the addition of 1 μg/mL puromycin (Gibco) into the culture media. After 4 days of selection, the cells were split across a 96-well plate into single colonies by serial dilution. Individual colonies were then grown and assessed for their degree of inducible RUNX1 repression.

#### Quantification of RUNX1 expression

To measure RUNX1 repression in the single colonies, each colony was split into two separate populations and grown in RPMI media supplemented with 10% FBS and 1% antibiotic-antimycotic and 1 μg/mL puromycin. In one of the groups, 1 μg/mL doxycycline (Thermo Fisher Scientific) was added to the media to induce expression of the dCas9-KRAB transgene. Both sets of cells were maintained at 200,000 cells/mL over the course of 3 days after the addition of the doxycycline. On day 3 the cells were pelleted, and RNA was extracted using a Qiagen RNeasy Mini Kit. Complementary DNA (cDNA) was synthesized from the RNA using the Protoscript II First Strand cDNA Synthesis Kit (New England BioLabs) per the manufacturer’s protocol, then diluted 1:4 with water. To quantify expression, qPCR was performed on the cDNA using a CFX Connect Real-Time PCR Detection System (Bio-Rad). For each sample, two sets of primers were used; a set used to quantify RUNX1 expression (Table S12) which was compared to the housekeeping gene GAPDH. The qPCR was carried out in a total volume of 10 μL containing 5 μL of iTaq Universal Sybr Green Master Mix (Bio-Rad), 2 μL of each primer (10 μM), and 1 μL of diluted cDNA. Thermal cycling conditions were 95°C for 2.5 min, followed by 40 cycles of 95°C for 10 s, then 60°C for 30 s. All samples were run in triplicate, and the RUNX1 expression was determined using the 2-delta delta CT method, by comparing to the GAPDH expression. The clone with the highest degree of RUNX1 repression was selected for use in the screen and subsequent experiments.

#### Sequencing screening

The K562 clonal cell line previously generated for repression of the endogenous RUNX1 protein was cultured in RPMI media supplemented with 10% FBS and 1% antibiotic-antimycotic. For the single-cell RNA sequencing screen, the cells were transduced with the pooled variant library at a low MOI of ~0.3 to ensure that each cell received a single construct. The viral transduction was performed by mixing the virus with media containing 8 μg/mL polybrene (Millipore). The cells were suspended in this media at a concentration of 2 million cells per mL and spun at 1000 G for 2 h at 33°C in a 12-well plate. The cells were then pelleted and resuspended in fresh media at a concentration of 400,000 cells/mL. 24 h after transduction, the media was again changed, and the cells were resuspended at 400,000 cells/mL. 48 h after transduction, the cell culture media was changed to media containing 1 μg/mL puromycin and 200 μg/mL hygromycin (Invitrogen) to select for transduced cells. At that time the cells were also split into two separate populations, and to one of the populations doxycycline was added daily at a concentration of 1 μg/mL to induce repression of the endogenous RUNX1. Throughout the duration of the screen, the media was changed each day, and the cells were maintained at a concentration of 400,000 cells/mL. The screening was conducted with two biological replicates with greater than 1 million cells in each condition to ensure greater than 1000-fold coverage of the library. At day 7 post transduction, a subset of the cells was processed with single-cell RNA sequencing, with the remainder of cells being maintained until day 14 for fitness screening.

For the bulk RNA sequencing and bulk ATAC sequencing screen, the cells were transduced with the twelve validation mutations separately. 48 h after transduction, 200 μg/mL hygromycin was used to select for transduced cells and 1 μg/mL doxycycline was used to induce repression of the endogenous RUNX1. The screen was conducted with three biological replicates with greater than 1 million cells in each condition. At day 7 post transduction, the cells were split into two groups, 1 million cells for bulk RNA-seq and 100,000 cells for bulk ATAC-seq.

#### Single-cell RNA sequencing library preparation

scRNA-seq experiments were performed with two replicates per condition (cells with and without doxycycline). Cells were first washed with a solution of PBS (Gibco) with 0.04% BSA (Gibco) by centrifuging the cells for 5 min at 300 G then resuspending them in the solution. After the wash, the cells were again centrifuged and resuspended in the same solution. The cells were filtered using a 40 μm cell strainer (VWR), and the concentration was determined using a manual hemacytometer (Thermo Fisher Scientific). The cells were then subjected to scRNA-seq (10X genomics, chromium single cell 3′ v3, with two reactions per replicate) aiming for a target cell recovery of 10,000 cells per library. The single-cell libraries were generated according to manufacturer’s protocols with the following conditions: 11 PCR cycles run during cDNA amplification and 10 PCR cycles run during library generation. The libraries were sequenced using Illumina NovaSeq platform. To genotype the cells with the variant, the barcode sequences were amplified off of the cDNA pool generated in the scRNA-seq protocol. The barcodes were amplified via PCR using OneTaq 2X Master Mix (New England BioLabs) in 100 μL reactions, each split across 5 PCR tubes (20 μL per tube). For each sample the reactions contained 5 μL of primers RX1_07 and the NEBNext Universal PCR Primer for Illumina (New England BioLabs) (10 μM), 6 μL of cDNA, 50 μL of OneTaq, and the rest filled with water. A thermal cycler was used to heat the sample to 95°C for 3 min, then 20 cycles of 98°C for 20 s, 65°C for 15 s, and 68°C for 45 s, followed by a final 5 min extension at 68°C. The PCR products were purified using AMPure XP Beads beads at a 0.8:1 bead:PCR reaction ratio. The second step of PCR was performed. Subsequently, a NEBNext Ultra RNA Library Prep Kit (New England BioLabs) was used to generate Illumina compatible sequencing libraries; this was done in a 50 mL reaction split across 5 PCR tubes (10 μL per tube) with 20 ng of the first step purified PCR product.

#### Library fitness screening

A fitness screen was also performed concurrently with the single-cell RNA sequencing screen. At days 2, 7, and 14 post-transfection, ~1 million cells were collected, and their genomic DNA was isolated via a Qiagen DNeasy Blood and Tissue Kit. Barcodes corresponding to each library element at each timepoint, and replicate were then amplified from the genomic DNA using OneTaq 2X Master Mix. The sequencing libraries were amplified in 50 μL reactions, each split across 5 PCR tubes (10 μL per tube). For each sample, the reactions contained 2.5 μL of primers A and B (10 μM), 6 μg of gDNA, 25 μL of OneTaq, with the rest filled with water. The thermal cycler was used to heat the sample to 95°C for 3 min, then 27 cycles of 98°C for 20 s, 65°C for 15 s, and 72°C for 45 s, followed by a final 5 min extension at 72°C. The PCR products were purified using AMPure beads at 0.8:1 bead:PCR reaction ratio. NEBNext Multiplexed Oligos for Illumina (New England BioLabs) were then used to index the samples, and the samples were sequenced on an Illumina NovaSeq platform to a depth of 2.5 million reads/sample.

#### Freezing for bulk RNA-seq

Cells for bulk RNA-seq were pelleted and the media aspirated. They were flash-frozen in liquid nitrogen and stored at −80°C.

#### Freezing for bulk ATAC-seq

Cells for bulk ATAC-seq were pelleted in a centrifuge at 1000 G for 5 min at 4°C, resuspended in cold PBS, and pelleted again. ATAC lysis buffer was made by mixing 100 μL 1M Tris-HCl pH 7.4, 20 μL 5 M NaCl, 30 μL 1M MgCl2, 100 μL 10% IGEPAL CA-630, and 9.75 mL water. The cells were lysed with the cold ATAC lysis buffer using 100 μL buffer per 100,000 cells and centrifuged at 1000 G for 10 min at 4°C. The supernatant was removed, and the cells were flash-frozen in liquid nitrogen and stored at −80°C.

#### Bulk RNA sequencing library preparation

Bulk RNA-seq experiments were performed with three replicates per condition. RNA was isolated from the cells using a Qiagen RNeasy Mini Kit according to the manufacturer’s protocols. Samples were prepared for bulk RNA-seq using the NEBNext Ultra II RNA Library Prep with Sample Purification Beads Kit (New England Biolabs) according to manufacturer’s protocols with the following conditions: 1 μg input RNA, library insert size = 200 nt. The bulk RNA-seq library was sequenced on an Illumina NovaSeq platform to a depth of 30 million reads/sample.

#### Bulk ATAC sequencing library preparation

Bulk ATAC-seq experiments were performed with three replicates per condition. Tagmentation buffer was prepared with 12.5 μL buffer, 9.75 μL H_2_O, 0.25 μL digitonin, and 2.5 μL Tn5 enzyme (Illumina) per sample. Each frozen cell pellet sample was resuspended in the tagmentation buffer and incubated at 37°C for 45 min. 1x volume 40mM EDTA was added to each sample. The tagmented samples were purified using AMPure XP Beads at a 2:1 bead:tagmentation reaction ratio. The samples were incubated with the beads at room temperature for 15 min, then placed on a magnetic rack to separate the beads from the supernatant, which was discarded. The beads were washed twice with cold 80% ethanol, and the purified DNA was eluted from the beads using Buffer EB (Qiagen).

The tagmented DNA was dual indexed using i5 and i7 barcodes, giving each sample a unique barcode combination. The DNA and barcodes were added to NEB Hi Fidelity 2x PCR Mix (New England BioLabs) and amplified using the following PCR cycle: 72°C for 7 min; 98°C for 30 s; then 10 cycles of 98°C for 10 s, 63°C for 30 s, and 72°C for 1 min; and cooling back down to 4°C. Double size selection was performed using AMPure XP Beads to select for the size of the final library. First, 0.55x volume AMPure Beads was added to each PCR reaction and incubated at room temperature for 15 min. The samples were placed on a magnetic rack and the supernatant transferred to new tubes, to which another 0.65x volume AMPure Beads were added (for a total of 1.2x volume PEG). The samples were incubated at room temperature for 15 min, the supernatant was discarded, and the beads were washed twice with cold 80% ethanol. DNA was eluted from the beads using Buffer EB and pooled together to make the final library for sequencing. The bulk ATAC-seq library was sequenced on an Illumina NovaSeq platform to a depth of 75 million reads/sample.

#### Western blot

K562 cells for each condition in Figures S2B and S2D were spun down at 300 rcf for 5 min and washed once with PBS. Cells were then resuspended in 100 μL of lysis buffer containing 1X RIPA Buffer (Cell Signaling Technology #9806), 0.1% SDS, and 1X protease inhibitor (Thermo Scientific #A32963). The samples were then spun at 21,000 rcf for 15 min and the soluble fraction was isolated. Protein concentration was then determined using the Pierce BCA Protein Assay kit (Thermo Fisher #23227), samples were diluted to 1 mg/mL with 4X Laemmli sample buffer (Bio-Rad #1610747) containing β-mercaptoethanol and were boiled for 10 min at 100 C. Using the Mini Trans-Blot Cell system (Bio-Rad), 20 μL of each condition was run on a 4–20% Mini-PROTEAN TGX gel (Bio-Rad) at 200V until completion. Protein transfer onto a PVDF membrane was performed at 70V for 120 min using the Trans-Blot system (Bio-Rad). Membranes were blocked using 5% non-fat milk in TBST (50 mM tris base, 150 mM NaCl, 0.1% Tween 20) for 60 min. Primary antibody incubations were performed with 5% BSA in TBST at 4°C for 16 h (RUNX1 sc-365644 Santa Cruz diluted 1:100; β-actin A1978 Sigma diluted 1:1000). Membranes were then washed for 10 min in TBST three times. Secondary antibody incubation was performed with anti-mouse IgG, HRP-linked antibody (Cell Signaling Technology #7076P2) diluted in 5% non-fat milk in TBST for 1 h at room temperature followed by three 10-min washes with TBST. Membranes were then developed using SuperSignal West Pico Plus Chemiluminescent Substrate (Thermo Fisher #34577).

For western blot regarding Figure 5H, whole cell lysate was extracted from K562 cells transduced with RUNX1 overexpression constructs with endogenous RUNX1 present. A total of 1 × 10^6^ cells were spun down at 500 rcf for 5 min and washed once with PBS. Cells were then resuspended in 100 μL of lysis buffer containing 0.05 M Tris pH 6.8, 10% glycerol, 2% SDS, 0.01 M DTT and boiled at 100°C for 10 min. All samples were immediately placed on ice for 5 min and 20 μL of 6x sample buffer consisting of 0.05 M Tris pH 6.8, 10% glycerol, 0.10 M DTT, 2% SDS, and 0.1% bromophenol blue was added to each sample. Using the Mini Trans-Blot Cell system (Bio-Rad), 10 μL of each sample was run on a 10% acrylamide gel (Fisher BP1408-1) at 90V for 15 min then 115V until completion. Protein transfer onto a PVDF membrane was performed at 280 mA for 90 min using the Trans-Blot system (Bio-Rad). Membranes were blocked using 5% non-fat milk in TBST (50 mM tris base, 150 mM NaCl, 0.1% Tween 20) for 30 min. Primary antibody incubations were performed in the same solution at 4°C for 16 h (RUNX1 sc-365644 Santa Cruz; β-actin A1978 Sigma). Membranes were then washed for 10 min in TBST three times. Secondary incubation was performed with Goat anti-mouse IRDye680 (926–68070 Li-Cor) for 1 h at room temperature followed by three 10-min washes with TBST. Membranes were scanned using a Li-Cor Odyssey device and analyzed using Li-Cor Image Studio Lite v 5.2.

#### scRNA-seq analysis

The single-cell RNA sequencing screen was performed for two conditions: one treated with doxycycline to induce repression of the endogenous RUNX1 (named ‘dox’ condition), and the other not treated (named ‘nodox’ condition). The screening was conducted with two biological replicates for each condition, and single-cell RNA sequencing was performed with two reactions per replicate, making a total of eight libraries: four containing cells treated with doxycycline (dox) and four not (nodox). Sequencing was run with a target cell recovery of 10,000 cells per library.

Sequencing reads in FASTQ format were aligned using the 10X Genomics Cell Ranger pipeline (version 3.1.0),^66^ to the human transcriptome GRCh38 (version GRCh38–3.0.0), resulting in a gene by cell matrix of UMI counts for each library. To assign one or more genotypes to each cell, the plasmid barcode reads were aligned to GRCh38 using BWA, and labeled with its corresponding cell and UMI tags as described in the SEUSS pipeline^12^ (https://github.com/yanwu2014/genotyping-matrices).

The UMI count matrices were processed using Seurat (version 4.1.0).^111^ Four dox and four nodox libraries were merged resulting in 86,120 cells and 21,153 genes, after removal of genes expressed in fewer than 3 cells. 44,418 cells not containing a genotype barcode, or containing more than one, were removed. To filter out low quality cells, we removed cells expressing fewer than 200 genes or more than 5000 genes. We also discarded cells that have over 20% of reads aligned to mitochondrial genes. Four perturbation variants (G138V, S145I, P157R, T161I) were excluded due to low cell counts (less than 10 cells for each condition), and one negative control was removed (G143G) due to a frameshift artifact occurred during the mutation library preparation, resulting in 40,522 cells corresponding to 112 remaining variants (20,878 cells for dox, and 19,644 cells for nodox condition).

The count matrix was log-normalized with the default scale factor of 10,000 and the top 2000 variable genes were identified to be used for downstream analyses. Mitochondrial or ribosomal genes were not included in the top 2000 variable gene list. We then applied a linear transformation on the count matrix to center and scale the expression of each gene. We assigned cell cycle scores to each cell based on its expression of G2/M and S phase markers and applied a linear model to regress out effects of cell cycle heterogeneity. We performed linear dimensionality reduction (PCA) on the scaled data using the top 2000 variable genes (Figure S3A).

#### T2 scores

In order to quantify the extent to which the expression profile of a variant deviates from the WT or LOF control variants, we used the Hotelling’s two-sample T-squared statistic (T2), a generalization of Student’s t-statistic that is used in a two-sample multivariate hypothesis testing.^71^ For this comparison, we employed the principal component space, using the top 20 principal components (PC) to compare matrices of cells × 20 PCs for each variant. We used the hotellings2 function from the spm1d python package to compute the test statistics, named here as T2 scores. For each variant, first we compared against cells overexpressing the WT variant (T2 scores (vs. WT)), then we compared against cells overexpressing the LOF variant (T2 scores (vs. LOF)). Higher scores indicate a higher deviation from the variant being compared.

Based on T2 scores, for each variant, cells with the endogenous RUNX1 repressed (dox) displayed higher deviation from the WT or LOF control variants overall, in comparison to the cells carrying the endogenous RUNX1 (nodox) (Figures S3B and S3C). Therefore, we decided to continue downstream analysis with dox condition cells only, corresponding to 20,878 cells with 20,389 genes. We repeated the previously described steps for log-normalization, identification of top 2000 variable genes, scaling, cell cycle effect regression, and PCA for these 20,878 dox cells.

#### Unsupervised clustering of single cells

Using the first 20 principal components, we clustered cells by first determining the nearest neighbors of each cell in the PCA space, and then by applying a modularity optimization algorithm that iteratively groups cells together with a resolution parameter of 0.3. We used UMAP, a non-linear dimensionality reduction technique,^133^ to visualize the three predicted unsupervised clusters where similar cells are placed together in low-dimensional space (Figures 2A, 2B, 2F, S5A, and S5B). Unsupervised clusters were confirmed not to result from cell cycle phase heterogeneity, or batch effects from merging of four dox libraries (Figures S5A and S5B).

We applied Fisher’s exact test to evaluate the enrichment or depletion of assigned phenotypes (Figure 2G), variant classes, or cell cycle phases (Figures S5C and S5D) in each cluster. A log odds ratio (log(OR))>0 indicates enrichment, while a log(OR) < 0 indicates depletion.

#### Unsupervised clustering of variants

For each variant, we computed the mean expression (log-normalized) of each of the top 2000 variable genes across all cells corresponding to the variant, resulting in an expression vector of size 2000 for each variant, representing its mean expression profile. This generated a count matrix of 112 variants by 2000 genes. We performed PCA on the count matrix, and using the first 20 principal components, we clustered variants by first determining the nearest neighbors of each variant in the PCA space, and then applying a modularity optimization algorithm that iteratively groups variants together with a resolution parameter of 0.8. We used UMAP to visualize the three predicted unsupervised clusters where similar variants are placed together in low-dimensional space (Figures 2C–2E).

We performed differential gene expression analysis between variants using DESeq2.^115^ First, differentially expressed genes between WT and LOF control variants (203 genes: FDR<0.05) were obtained. Next, each hypomorphic variant was compared against the WT control variant separately, and genes that were differentially higher or lower expressed in at least one hypomorphic variant against WT (141 genes: FDR<0.05) were extracted. Then, the same procedure was performed against the LOF control variant (232 genes: FDR<0.05) (Figure S6A).

#### Fitness analysis

To calculate fitness effects from genomic DNA reads, we first aligned reads to mutation barcodes (MagECK^110^) and counted the number of reads corresponding to each mutation for each replicate at each timepoint (days 2, 7 and 14 post transduction), resulting in a mutation by samples read counts matrix. We normalized read counts for each sample by dividing each column by its sum. We then divided read counts of each sample by the counts at day 2 post transduction, and log2 transformed it to obtain a measurement to represent fitness effects for each mutation and sample. We averaged fitness measurements from the two biological replicates taken at day 14 (Figure 2I) to compute mean fitness scores (Figure 3D).

#### Hierarchical variant clustering

We also hierarchically clustered variants based on Pearson correlation of their mean gene expression profiles using the top 2000 variable genes. We ordered the leaves of the resulting dendrogram by increasing T2 scores obtained from comparison to the WT variant. To obtain discrete cluster assignments, we cut the dendrogram based on visual inspection, obtaining three main variant clusters that largely agree with WT-like, hypomorphic and LOF-like annotations (only 1 variant difference for the hypomorphic/LOF-like separation). We further cut the dendrogram of the middle cluster, representing hypomorphic variants, into three sub-clusters: named as hypomorphic-I, hypomorphic-II, and hypomorphic-III.

#### Hierarchical gene clustering

To determine genes whose expression is impacted by variants, we hierarchically clustered genes based on Manhattan distance between them using mean gene expression profiles of variants, resulting in gene groups with various expression profiles across variant clusters.

#### Cell state analysis

Cell states can be described by activities of coordinated gene expression programs.^134–138^ We applied a non-negative matrix factorization algorithm (CoGAPS^72^ on the expression matrix of the top 2000 variable genes of 14,217 cells harboring perturbation variants or WT or LOF control constructs, using default parameters, which produced a gene by pattern (2000 × 7) and a pattern by cell (7 × 14,217) matrix. Using the pattern by cell matrix, we hierarchically clustered the top 2000 variable cells into 7 clusters which roughly corresponds to the 7 identified patterns (Figure S8A) and applied a Fisher’s exact test to evaluate the enrichment or depletion of variant phenotypic annotations for each cluster (Figure S8B). Using the gene by pattern matrix, we assigned non-overlapping gene markers for each pattern by distributing genes into patterns with the lowest ranking.

#### MLL dataset

Patient samples sent to the Munich Leukemia Laboratory (MLL) for routine diagnostic workup between August 2005 and March 2023 and that were diagnosed with AML were queried for missense mutations in RUNX1. AML diagnoses were based on cytomorphology, immunophenotype, cytogenetics, and molecular genetics following gold standard practices. All patients gave their written informed consent for scientific evaluations. The study was approved by the Internal Review Board and adhered to the tenets of the Declaration of Helsinki. In total, 716 individuals from the MLL cohort carried 1 or more missense mutations in the Runt domain (amino acid positions 50–177), totaling 772 mutations. Mutations were defined with respect to the ENST00000344691 transcript of RUNX1.

#### Germline variants

529 unique RUNX1 missense variants were obtained from the ClinVar database^87^ (on 02/05/2024, for transcript NM_001001890), along with their clinical significance annotations (‘germline classification’ column). We divided them into three categories based on clinical significance: benign (if annotated as ‘benign’ or ‘likely benign’), pathogenic (if annotated as ‘pathogenic’ or ‘likely pathogenic’), or VUS (if annotated as ‘uncertain significance’). Only two non-Runt domain variants had ‘conflicting classifications of pathogenicity’. Among 148 Runt domain variants, 24 overlap with our library, of which one is annotated as benign, 7 as pathogenic and 16 as VUS. Among the remaining 124 not present in our library, one is annotated as benign, 20 as pathogenic, and 103 as VUS.

#### Reference population variation

521 unique RUNX1 missense variants were obtained from the gnomAD database^88^ (version 4.0.0, on 02/05/2024, for transcript ENST00000344691), 89 located in the Runt domain, of which 12 overlap with our library. 9 of the 12 also overlap with ClinVar variants. Of the remaining 77 not present in our library, 36 are also observed in ClinVar.

#### Predicting variant transcriptomic effects

To generate a binary classification task, we divided the 79 RUNX1-perturbing variants in our library into a positive/functional (38 functional variants: 24 LOF-like and 14 hypomorphic) vs. negative/WT-like class (41 WT-like variants). First, we obtained 85 features for each variant from the SNVBox database^92^ describing substitution effects on amino acid biophysical properties, evolutionary conservation of variant sites, local sequence biases, and site-specific functional annotations. Then, we performed a 60–40 random split on the dataset, to generate a training (*n* = 49: 24 functional, 25 WT-like) and a test set (*n* = 30: 14 functional, 16 WT-like) with balanced ratios of functional and WT-like class variants.

We trained a Random Forest classifier (n_estimators = 1000, max_features = ‘sqrt’) on the training set using the scikit-learn Python package and tested on the test set. The classifier score (between 0 and 1) represents the percentage of decision trees that classify a mutation as functional/positive. Receiver Operator Characteristic (ROC) and Precision–Recall curves (PR) were constructed from the classifier scores and the AUC statistic was used as a measure of classifier performance.

Next, we trained another Random Forest classifier using the entire dataset of 79 perturbation variants and predicted transcriptomic effect labels for all remaining possible missense mutations on the RUNX1 protein (*n* = 2594). We used the positive (5 core mutations) and negative (7 predicted neutral mutations) missense control variants in our RUNX1 mutation library as a validation set. We used a 0.5 score cutoff to designate predictions as functional (score>0.5) vs. WT-like (score<0.5).

To further assess classifier performance, we created a high confidence dataset (*n* = 184) using Runt domain missense mutations obtained from the COSMIC, MLL, ClinVar and gnomAD databases. Pathogenic class (*n* = 110) consists of cancer mutations (COSMIC or MLL) with frequency>1 or ClinVar mutations with ‘pathogenic’ or ‘likely pathogenic’ clinical significance annotations. Neutral class (*n* = 74) consists of ClinVar mutations with ‘benign’ or ‘likely benign’ clinical significance annotations or gnomAD mutations without ‘pathogenic’ or ‘likely pathogenic’ ClinVar annotations. This dataset excludes our 79 perturbation library variants used in the classifier training.

#### Selecting variants for validation with bulk sequencing

Ten hypomorphic variants showing the largest deviation from control conditions based on mean of T2_WT_ and T2_LOF_ scores were selected: two hypomorphic-I (N82I, P156R), seven hypomorphic-II (L62P, L94P, G95R, V97D, G100V, V137D, I166S) and one hypomorphic-III (R118G). L94P was removed for being predicted to target similar protein interactions as G95R, and a LOF-like variant (V159D) predicted to be involved in RUNX1-CBFB binding was added. WT and LOF control variants were included bringing the total number of variants chosen for bulk sequencing to 12. Variant relative protein expression was calculated by dividing the raw expression values of RUNX1 variants to corresponding β-actin expression, used as a control, and then normalized to GPF/LOF control variant expression (Figures 5H and 5I).

#### Bulk RNA-seq analysis

Sequencing reads in FASTQ format were aligned to the human transcriptome GRCh38 (Gencode v30 - GRCh38.p12) using STAR (version 2.7.1a).^113^ RSEM (version 1.3.1) is used to calculate read counts for each sample and replicate (‘rsem-calculate-expression’ command), and to generate a gene by sample matrix (‘rsem_generate_data_matrix’ command) of the raw counts (‘expected_count’ column). Starting with 57,535 gene features, we removed genes with less than 10 reads in total across all the samples, along with mitochondrial and ribosomal genes, resulting in 18,646 remaining genes.

We first normalized raw counts using the variance stabilizing transformation, which transforms counts on the log2 scale and normalizes with respect to library size.^115^ We removed two outlier samples (replicates 2 of samples with N82I and V137D mutations) identified based on expression profiles of top 2000 variable genes (Figure S12) and removed batch effects between replicates using the limma package.^114^ For visualization purposes, we averaged gene expression across replicates for each sample. To validate variant clustering results obtained from scRNA-seq here in the bulk setting, we used top 2000 variable genes obtained from the scRNA-seq analysis, to perform PCA and hierarchical clustering of samples and genes based on Manhattan distance. We ordered the leaves of the resulting sample dendrogram by increasing T2 scores obtained from the scRNA-seq analysis by comparison to the WT variant. The same analysis was also performed using all replicates instead of their means (Figure S10).

Differential expression analysis between samples was performed with DESeq2.^115^ Each hypomorphic variant was compared against the WT and LOF control variants separately, and genes that were differentially (FDR<0.05) upregulated (202 genes) or downregulated (89 genes) in at least one hypomorphic variant against both WT and LOF controls were extracted.

RNA expression coverage tracks (bigWig files) were generated from BAM format using deepTools bamCoverage.^116^

#### Bulk ATAC-seq analysis

Sequencing reads in FASTQ format were aligned to the human transcriptome GRCh38 and processed using the nf-core/atacseq pipeline (version 1.2.2),^117^ built using Nextflow (version 22.04.0), in conjunction with Singularity. The command used is ‘nextflow run nf-core/atacseq -r master -name “run/name” -profile “singularity” -work-dir “work/directory/path” -params-file “params/file/path” –genome GRCh38 –narrow_peak true’, with default parameters. First, fastq files from two ATAC-seq runs were merged with “cat” command for each read (reads 1 and 2 for paired-end data) of each replicate (three biological replicates) of each sample (12 samples); and the pipeline was run on the merged FASTQ files. Briefly, the pipeline performs adapter trimming using Trim Galore! (https://www.bioinformatics.babraham.ac.uk/projects/trim_galore/), read alignment with BWA,^139^ filtering with SAMtools^119^ (e.g., removal of mitochondrial reads), BEDTools,^122^ BamTools,^140^ Pysam (https://github.com/pysam-developers/pysam), and picard (https://broadinstitute.github.io/picard/), normalized coverage track generation with BEDTools and bedGraphToBigWig,^120^ genome-wide enrichment with deepTools,^116^ peak calling with MACS2^121^ (narrow peaks), and quality control and statistics reporting with MultiQC.^141^

Coverage tracks were further processed with AtacWorks,^118^ which uses a deep learning model trained on high quality ATAC-seq data to remove background noise. ATAC-seq data from K562 cells was obtained from the ENCODE data portal^142^ (experiment ENCSR868FGK). To generate a model of noisy data, the aligned reads files from replicate 1 (ENCFF534DCE) were subsampled to about 20 million reads with SAMtools view,^119^ and converted to bigWig format with deepTools bamCoverage.^116^ The resulting bigWig file and the bigWig for the entirety of replicate 1 (ENCFF670QXU) were provided to AtacWorks as the noisy versus clean data to train the model.

The two highest quality replicates for each RUNX1 variant (bigWig files) were denoised using the trained AtacWorks model and combined with UCSC bigWigMerge.^120^ Peaks were called on the summed files using MACS2 callpeak^121^ and were compared against the ENCODE K562 ATAC data and filtered as follows.

For the wild-type sample:

1. denoised peaks that were not observed in the undenoised peak set or in the ENCODE peak set were marked as noise and removed,
2. peaks that were seen in the denoised bigWig track but lost during peak calling and that were also observed in either the undenoised or the ENCODE peak set were rescued.

For the other samples:

1. denoised peaks that were not observed in the undenoised peak set or in the ENCODE peak set were marked as noise and removed,
2. peaks that were seen in the denoised bigWig track but lost during peak calling and that were also observed in the ENCODE peak set were rescued as “wild type” peaks,
3. peaks that were seen in the denoised bigWig track but lost during peak calling and that were also observed in the undenoised peak set were rescued as “mutation” peaks.

The filtered peaks were merged with BEDTools merge^122^ to generate the final denoised consensus peak set. On the consensus peak set, read counts were obtained with featureCounts.^123^ Using HOMER,^124^ enriched motifs were identified (findMotifsGenome function) and filtered for Runt domain motifs (5 motifs total) to represent RUNX1 DNA binding sites. Consensus peaks were annotated with genomic features and Runt motifs (within 1000 base pairs) using HOMER (annotatePeaks function). They were also annotated with ChromHMM states^143^ using a 25-state model for K562 cells obtained from the Roadmap Epigenomics Project (https://egg2.wustl.edu/roadmap/web_portal/). Peaks that overlap with Hi-C loops^144^ were identified with BEDTools pairtobed^122^ using Hi-C chromatin loop data for K562 cells obtained from NCBI GEO (GSM1551620).

We identified peaks as promoters if located within 1 kbp downstream and 100 bp upstream of the transcription start site (TSS) of a gene. We identified peaks as enhancers if annotated with a ChromHMM enhancer state. We filtered both promoter and enhancer peaks that contain Runt motifs, to study genes regulated by RUNX1. Genes associated with each enhancer peak were identified using the Hi-C chromatin loops.

RNA expression and DNA accessibility coverage tracks, ChromHMM states and Hi-C loops were visualized using CoolBox.^125^

### QUANTIFICATION AND STATISTICAL ANALYSIS

All computational analyses were performed in Python or R. Correlations were evaluated using the Pearson correlation coefficient. Odds ratios were calculated with Fisher’s exact test. Distributions were compared with Mann–Whitney U test. Multiple testing correction is applied where applicable using the Benjamini-Hochberg method.

Gene set overrepresentation analysis is performed using the Gene Ontology (GO) biological process terms (2021) or Reactome pathways (2022), with the EnrichR package.^112^

## Supplementary Material

1

2

3

4

5

6

7

8

9

10

## Figures and Tables

**Table 1.** Validation variants selected for bulk RNA-seq and ATAC-seq

Variant	Class	Phenotype	Tumors, *n*

L62P	perturbation	hypomorphic-2	0
N82I	perturbation	hypomorphic-1	0
G95R	perturbation	hypomorphic-2	1
V97D	perturbation	hypomorphic-2	0
G100V	perturbation	hypomorphic-2	1
R118G	perturbation	hypomorphic-3	3
V137D	perturbation	hypomorphic-2	0
P156R	perturbation	hypomorphic-1	0
V159D	perturbation	LoF-like	1
I166S	positive	hypomorphic-2	0
LoF	LoF	LoF-like	0
WT	WT	WT-like	0

Variant classes, phenotypic designations, and frequency in human tumors (COSMIC or MLL).

**KEY RESOURCES TABLE:** 

REAGENT or RESOURCE	SOURCE	IDENTIFIER

Antibodies		

RUNX1 Antibody (A-2), mouse monoclonal	Santa Cruz Biotechnologies	Cat#sc-365644; RRID:AB_10843207
Anti-β-Actin Antibody (AC-15), mouse monoclonal	Millipore Sigma	Cat#A1978; RRID:AB_476692
Anti-Mouse IgG, HRP-linked Antibody	Cell Signaling Technology	Cat#7076P2; RRID:AB_330924
IRDye 680RD Goat anti-Mouse IgG Secondary Antibody	Li-Cor Bio	Cat#926-68070; RRID:AB_10956588

Bacterial and virus strains		

Stbl3	Thermo Fisher	Cat#C737303

Chemicals, peptides, and recombinant proteins		

NheI-HF	New England Biolabs	Cat#R3131S
SalI-HF	New England Biolabs	Cat#R3138S
AflII	New England Biolabs	Cat#R0520S
Gibson Assembly Master Mix	New England Biolabs	Cat#E2611S
KAPA HiFi HotStart ReadyMix	Roche	Cat#KK2601
OneTaq 2X Master Mix	New England Biolabs	Cat#M0482S
AMPure XP Beads	Beckman Coulter	Cat#A63880
DMEM	ThermoFisher Scientific	Cat#10566016
.05% Trypsin-EDTA	Gibco	Cat#25300062
OptiMEM	ThermoFisher Scientific	Cat#31985062
RPMI Medium 1640	Gibco	Cat#11875-093
FBS	Gibco	Cat#A52568
Anti-anti	Gibco	Cat#15240-062
Lipofectamine 2000	ThermoFisher Scientific	Cat#11668030
Doxycycline hyclate	Sigma	Cat#D5207
Hygromycin	Invitrogen	Cat#10687010
Puromycin	Gibco	Cat#A11138-03
Polybrene	Millipore-Sigma	Cat#TR-1003-G
PBS, pH 7.4	Gibco	Cat#10010023
Tn5	Illumina	Cat#20034198
RIPA Buffer	Cell Signaling Technologies	Cat#9806
Pierce Protease Inhibitor Tablets	ThermoFisher Scientific	Cat#A32963
4X Laemmli Sample Buffer	Bio-Rad	Cat#1610747
4–20% Mini-PROTEAN TGX gel	Bio-Rad	Cat#4561094
10X Tris/Glycine/SDS Buffer	Bio-Rad	Cat#1610732
Nonfat Dry Milk	Apex Bioresearch	Cat#20-241
TBST	Cell Signaling Technology	Cat#9997
BSA, Fraction V, Fatty Acid-Free	Millipore Sigma	Cat#126575
SuperSignal West Pico Plus	ThermoFisher Scientific	Cat#34577
Chemiluminescent Substrate		
Carbenicillin	Teknova	Cat#C2199
iTaq Universal SYBR Green Master Mix	Bio-Rad	Cat#1725120

Critical commercial assays		

Pierce BCA Protein Assay kit	ThermoFisher Scientific	Cat#23227
RNeasy Mini Kit	Qiagen	Cat#74104
DNeasy Blood and Tissue Kit	Qiagen	Cat#69504
Protoscript II First Strand cDNA Synthesis Kit	New England BioLabs	Cat#E6560S
QIAquick PCR Purification Kit	Qiagen	Cat#28104
Nucleofector Solution Set SF	Lonza	Cat#PBC2-00675
Chromium Single Cell 3’ v3	10X Genomics	Cat# PN-1000128
NEBNext Ultra RNA Library Prep Kit	New England Biolabs	Cat#E7530

Deposited data		

Raw data: scRNA-seq	This paper	SRA: PRJNA1033389
Raw data: bulk RNA-seq	This paper	SRA: PRJNA1121326
Raw data: ATAC-seq	This paper	SRA: PRJNA1121327
RUNX1-CBFB-DNA structures	Protein DataBank	PDB: 1ljm, 1e50, 1h9d
Human reference genome NCBI build 38, GRCh38-3.0.0	Genome Reference Consortium	http://www.ncbi.nlm.nih.gov/projects/genome/assembly/grc/human/
ATAC-seq data for K562	ENCODE	ENCSR868FGK
ChromHMM states for K562	Roadmap Epigenomics Project	https://egg2.wustl.edu/roadmap/web_portal/
Hi-C chromatin loop data for K562	NCBI	GEO: GSM1551620

Experimental models: Cell lines		

K-562	ATCC	Cat#CCL-243; RRID:CVCL_0004
HEK293T	ATCC	Cat#CRL-3216; RRID:CVCL_0063

Oligonucleotides		

RUNX1 library variants	Twist Bioscience	https://www.twistbioscience.com
Primers for qPCR, Sanger Sequencing, & oligopool amplification, see Table S10	This Paper	N/A
NEBNext Multiplex Oligos for Illumina	New England Biolabs	Cat#E7335S

Recombinant DNA		

EF1a_mCherry_P2A_Hygro_Barcode	(Parekh et al.)^12^	Addgene #120426
PB-TRE-dCas9-VPR	(Chavez et al.)^108^	Addgene #63800
pHR-SFFV-KRAB-dCas9-P2A-mCherry	(Gilbert et al.)^109^	Addgene #60954
pMD2.G	N/A	Addgene #12259
pCMV delta R8.2	N/A	Addgene #12263

Software and algorithms		

Code related to analyses of RUNX1 mutations	This paper	https://github.com/cartercompbio/RUNX1_SEUSS; https://doi.org/10.5281/zenodo.11580866
CellRanger 3.1.0	10X Genomics	https://support.10xgenomics.com
genotyping-matrices	(Parekh et al.)^12^	https://github.com/yanwu2014/genotyping-matrices
MAGeCK	(W. Li et al.)^110^	https://sourceforge.net/p/mageck/wiki/Home/
PRISM	(Baspinar et al.)^60^	https://cosbi.ku.edu.tr/prism/
VEST	(Carter et al.)^62^	http://www.cravat.us/CRAVAT/
FoldX	(Schymkowitz et al.)^63^	http://foldx.crg.es/
Seurat 4.1.0	(Macosko et al.)^111^	https://satijalab.org/seurat/
CoGAPS	(Fertig et al.)^72^	http://www.bioconductor.org/packages/release/bioc/html/CoGAPS.html
EnrichR	(Kuleshov et al.)^112^	https://github.com/wjawaid/enrichR
STAR 2.7.1a	(Dobin et al.)^113^	https://github.com/alexdobin/STAR
RSEM 1.3.1	N/A	http://deweylab.biostat.wisc.edu/rsem/
limma	(Ritchie et al.)^114^	http://bioinf.wehi.edu.au/limma/
DESeq2	(Love et al.)^115^	https://bioconductor.org/packages/release/bioc/html/DESeq2.html
deepTools	(Ramirez et al.)^116^	https://deeptools.readthedocs.io/en/develop/
nf-core/atacseq 1.2.2	(Ewels et al.)^117^	https://github.com/nf-core/atacseq
AtacWorks	(Lal et al.)^118^	https://github.com/NVIDIA-Genomics-Research/AtacWorks
SAMtools	(H. Li et al.)^119^	http://htslib.org/
UCSC bigWigMerge	(Kent et al.)^120^	http://genome.ucsc.edu/
MACS2	(Zhang et al.)^121^	https://pypi.org/project/MACS2/
BEDTools	(Quinlan et al.)^122^	https://github.com/arq5x/bedtools2
featureCounts	(Liao et al.)^123^	http://bioinf.wehi.edu.au/featureCounts/
HOMER	(Heinz et al.)^124^	http://homer.ucsd.edu/
CoolBox	(Xu et al.)^125^	https://github.com/GangCaoLab/CoolBox

Other		

STRING v9.1	(Szklarczyk et al.)^126^	http://string.embl.de/
COSMIC	(Tate et al.)^64^	http://cancer.sanger.ac.uk/cancergenome/projects/cosmic/
MLL	Munich Leukemia Laboratory	https://www.mll.com
ClinVar	(Landrum et al.)^87^	https://www.ncbi.nlm.nih.gov/clinvar/
gnomAD 4.0.0	(Chen et al.)^88^	http://gnomad.broadinstitute.org/
SNVBox	(Wong et al.)^92^	https://chasmplus.readthedocs.io/en/latest/installation.html#snvbox-database-mysql

## Highlights

- scRNA-seq profiling of 115 variants at protein interfaces of the RUNX1 transcription factor
- Clustering identifies transcriptionally distinct groups and uncovers hypomorphic variants
- Hypomorphic variants alter gene programs associated with cytokine and growth factor signaling
- scRNA-seq-based assay informs reclassification of variants of uncertain significance
